# Seasonal and altitudinal changes of culturable bacterial and yeast diversity in Alpine forest soils

**DOI:** 10.1007/s00792-016-0874-2

**Published:** 2016-09-12

**Authors:** Luís França, Ciro Sannino, Benedetta Turchetti, Pietro Buzzini, Rosa Margesin

**Affiliations:** 1 Institute of Microbiology, University of Innsbruck, Technikerstrasse 25, 6020 Innsbruck, Austria; 2 Department of Agricultural, Food and Environmental Sciences, Industrial Yeasts Collection DBVPG, University of Perugia, Borgo XX Giugno 74, 06121 Perugia, Italy

**Keywords:** Psychrophiles, Culturable bacteria and yeasts, Alpine soils, 16S rRNA gene, 26S rRNA gene, ITS1&2, Bacterial OTUs, Yeast species

## Abstract

The effect of altitude and season on abundance and diversity of the culturable heterotrophic bacterial and yeast community was examined at four forest sites in the Italian Alps along an altitude gradient (545–2000 m). Independently of altitude, bacteria isolated at 0 °C (psychrophiles) were less numerous than those recovered at 20 °C. In autumn, psychrophilic bacterial population increased with altitude. The 1194 bacterial strains were primarily affiliated with the classes *Alpha*-, *Beta*-, *Gammaproteobacteria*, *Spingobacteriia* and *Flavobacteriia.* Fifty-seven of 112 operational taxonomic units represented potential novel species. Strains isolated at 20 °C had a higher diversity and showed similarities in taxa composition and abundance, regardless of altitude or season, while strains isolated at 0 °C showed differences in community composition at lower and higher altitudes. In contrast to bacteria, yeast diversity was season-dependent: site- and altitude-specific effects on yeast diversity were only detected in spring. Isolation temperature affected the relative proportions of yeast genera. Isolations recovered 719 strains, belonging to the classes *Dothideomycetes*, *Saccharomycetes*, *Tremellomycetes* and *Mycrobotryomycetes.* The presence of few dominant bacterial OTUs and yeast species indicated a resilient microbial population that is not affected by season or altitude. Soil nutrient contents influenced significantly abundance and diversity of culturable bacteria, but not of culturable yeasts.

**Electronic supplementary material:**

The online version of this article (doi:10.1007/s00792-016-0874-2) contains supplementary material, which is available to authorized users.

## Introduction

Soil microbial community structure, composition and function depend on an array of biotic and abiotic parameters, such as soil physicochemical characteristics, above ground vegetation, nutrient availability and environmental conditions, including macroclimate and microclimate, which in turn affect soil organic matter storage and turnover and biogeochemical cycling (Bossio et al. 1998; Rousk et al. 2010; Brockett et al. 2012; Urbanová et al. 2015). Our knowledge on how these complex interactions occur has been slowly increasing through recent years. Some studies have been focused on the effect of climate on the diversity and activity of soil prokaryotic and eukaryotic microbial communities with regard to latitude, but insufficient attention has been given to the effects of altitude (Vishniac 2006; Yergeau et al. 2007; Wu et al. 2009). The drastic variation of biotic and abiotic factors along altitude over short geographical distances have indicated that mountain areas can be used as a model to examine the stability of an ecosystem under environmental and climate changes (Diaz et al. 2003; Margesin et al. 2014). Furthermore, temperature strata and vegetation zonation along gradients of altitude are analogous counterparts of the latitude climatic zones since temperature decreases with increasing altitude and latitude (Kuhn 2008).

Our previous studies indicated a decrease of microbial abundance and activity, an increase of the ratio Gram-negative/Gram-positive bacteria and an increase of the relative proportion of culturable psychrophilic heterotrophic bacteria [with a dominance of *Proteobacteria* and a decrease of the *Cytophaga*–*Flavobacterium*–*Bacteroides* (CFB) group] with altitude due to increasing environmental harshness (Margesin et al. 2009, 2014). Other studies supported these conclusions and reported that major microbial structure changes across altitude gradients resulted in a decrease of bacterial and fungal diversity and in a loss of functional activity (Uchida et al. 2000; Lipson 2007; Xu et al. 2015). Inverse correlations between bacterial community abundance and altitude were also found in Chinese and Indian mountain slopes (Ma et al. 2004). Culture-independent approaches revealed a dominance of *Betaproteobacteria* at higher altitudes in western Himalayas, India, while *Gammaproteobacteria*, *Bacteroidetes* and lower proportions of Gram-positive bacteria (mainly *Firmicutes*) dominated the culturable community (Gangwar et al. 2009). However, others concluded that microbial diversity was not significantly altered across a montane elevational gradient in eastern Peru, using a barcoded pyrosequencing procedure, in opposition to the drastic changes observed for plant and animal taxa (Fierer et al. 2011). The same finding was corroborated by studies that described stable microbial community composition in tundra soils of Finnish Lapland and in elevations on Changbai Mountain in Asia along an increase of altitude as long as the soil pH was similar (Männistö et al. 2007; Shen et al. 2013). In contrast to bacteria, no studies on the impact of altitude on culturable yeast biodiversity in Alpine soils are available. The sole comparable study was carried out in Antarctic Dry Valleys, where fungal assemblages resulted to be dominated by ascomycetous yeasts (Yung et al. 2014).

Alpine soil environments are characterized by dramatic seasonal shifts in physical and biochemical properties along altitude gradients. Microbial communities may be shaped seasonally as response to the large and frequent (diurnal) temperature fluctuations, regular soil freeze–thaw and wet–dry events (Nemergut et al. 2005; Margesin 2012). Studies on the effect of seasons on diversity and abundance of alpine soil bacterial communities in the Colorado Rocky Mountains reported major changes in bacterial structure through seasons. *Acidobacterium* and CFB divisions were most abundant in spring, while *Verrucomicrobium* division and *Betaproteobacteria* dominated the uncultured community in summer (Lipson and Schmidt 2004). *Alphaproteobacteria* were equally abundant in all seasons. The majority of the cultured microorganisms affiliated with uncultured sequences that belonged mainly to the *Gamma*-, *Beta*- and *Alphaproteobacteria*, *Actinobacteria* and to the CFB group (Lipson and Schmidt 2004). In forest soils, a high microbial metabolic versatility and capability to adapt to climatic changes associated with seasonality was reported (Ma et al. 2004; Giri et al. 2007; Männistö et al. 2007; Rasche et al. 2011; Fierer et al. 2011; Shen et al. 2013). All these investigations agree that altitude per se is not the driving force to promote changes in the microbial structure, instead a wider range of factors, such as the temperature and precipitation regimes that may indirectly control soil carbon input by influencing plant community and primary production, has to be considered.

In Alpine environments, increasing temperature associated with global warming is expected to cause an upward migration of vegetation zones. This may alter the composition of vegetation as well as the quantity and quality of plant litter, which in turn affects microbial community composition and functioning (Gavazov 2010). Forests are expected to face significant pressures in the future from climate change. Up-to-date reports describing the variation of both prokaryotic and eukaryotic microbial communities in Alpine forest soils due to altitudinal and seasonal effects are very scarce, although such studies may help to explain the physiological characteristics of the microbial community as a whole as well as adaptation processes to fluctuating environmental conditions.

At low temperatures, which prevail at high altitudes and in seasonally cold periods, the activity of psychrophilic microorganisms is required to ensure decomposition processes of organic matter and to provide nutrients to plants. These microorganisms are characterized by their ability to grow well and to display high metabolic activities at low temperatures down to and even significantly below the freezing point of water (Margesin and Miteva 2011). The understanding to what extent psychrophilic communities are adapted to cold conditions and how they respond to temperature changes can shed light on how climate change will impact high altitude regions.

It is recognized that culture-dependent methods underestimate the abundance of microbial cells in environmental samples or that microorganisms isolated on culture media are rarely representative or functionally significant (Rappé and Giovannoni 2003). However, it has also been demonstrated that culturomics have the potential to retrieve both abundant and rare taxa and permit to obtain additional information about the physiological and metabolic state of the microbiota present in environmental samples (Pedrós-Alió 2006).

In our previous studies, we examined microbial activity, abundance and community structures in soils collected from four forest sites in the Italian Alps along an altitude gradient ranging from 545 to 2000 m (tree line) above sea level (Siles and Margesin 2016; Siles et al. 2016). These studies showed altitude-related shifts in soil microbial communities and indicated environmental and soil nutrient conditions prevailing at each site as driving factors. However, due to the application of non-culture-based techniques, the evaluation of altitudinal and seasonal impacts of psychrophilic and mesophilic microorganisms was not possible. Therefore, it was the objective of this study to examine the effects of altitude and season on the diversity of culturable heterotrophic bacterial and yeast communities in these soils collected in spring and autumn. To evaluate population shifts between psychrophiles and mesophiles as a result of the effect of altitude and/or season, we compared bacterial and yeast communities at two different isolation temperatures.

## Materials and methods

### Description of the study sites

The investigated four sampling sites represent widely distributed and forestally significant forest types in South Tyrol in the Italian Alps. The sites (M, K, R, S) are located across an altitudinal gradient from 545 to 2000 m above sea level (a.s.l.), representing a climosequence, including submontane, montane, subalpine, and alpine vegetation zones, respectively. All sites were SW-exposed and contained the same bedrock. A detailed description of each site has previously been reported (Siles and Margesin 2016; Siles et al. 2016). The characteristics of the studied sites are summarized in Table 1. Soils from all four sites were acidic and carbonate-free. Altitude had a significant (*P* < 0.001) effect on soil physicochemical properties; soil organic matter (SOM), total organic carbon (TOC) and other nutrients increased significantly with altitude. Nutrient contents in autumn were increased compared to spring. In both seasons, contents of SOM and soil nutrients (TOC, N, P, K, Mg) were lowest at the submontane site M and highest at the alpine site S (Table 2), which resulted in a significant increase in microbial activities at higher altitudes, contrary to the assumption that colder climate are limiting (Siles et al. 2016).

**Table 1:** Characteristics of the investigated forest sites (Siles and Margesin 2016; Siles et al. 2016)

Characteristics	M	K	R	S
Location	Kleiner Priol (Montiggl)	Klobenstein (Ritten)	Kleebach (Ritten)	Schwarzsee-spitze (Ritten)
Coordinates	N46°25′36.8″ E11°17′48.6″	N46°32′38.1″ E11°28′16.1″	N46°35′16.2″ E11°26′4.9″	N46°35′21.4″ E11°27′2.4″
Altitude (m a.s.l.)	545–570	1175–1200	1724–1737	1965–2000
Exposition	SW	SW	SW	SW
Mean annual precipitation (mm)	900	950	1000	1050
Mean annual air temperature (°C)	11.0	7.4	4.0	2.4
Mean annual soil temperature (°C)^b^	9.8 b^b^	9.3 b	4.3 a	3.9 a
Minimum annual soil temperature (°C)^b^	6.5 b	5.5 b	1.9 a	1.1 a
Maximum anual soil temperature (°C)^a^	13.5 b	12.7 b	6.1 a	6.2 a
Altitudinal vegetation belt	Submontane	Montane	Subalpine	Alpine
Vegetation	Mixed deciduous forest	Mixed deciduous forest	Coniferous forest	Tree line
Dominant plant species	*Quercus pubescens*, *Quercus robur*, *Fraxinus ornus*, *Pinus sylvestris*, *Ostrya carpinifolia*	*Fagus sylvatica*, *Pinus sylvestris*, *Picea abies*, *Larix decidua*	*Picea abies*, *Pinus cembra*, *Larix decidua*, *Vaccinium myrtillus*	*Pinus mugo*, *Pinus cembra*,*Picea abies*, *Rhododendron ferrugineum*
Bedrock	Rhyolite (quartz-porphyry)	Rhyolite (quartz-porphyry)	Rhyolite (quartz-porphyry)	Rhyolite (quartz-porphyry)
Soil type	Dystric cambisol	Dystric cambisol	Haplic podsol	Haplic podsol

*a.s.l.* above sea level

^a^Different letters in a row denote significant differences (*P* < 0.05) between sites

**Table 2:** Soil physicochemical properties at the investigated sites in spring and autumn (Siles et al. 2016)

Properties	Site M	Site K	Site R	Site S
Spring				
pH (CaCl_2_)	4.5^b^	4.1^b^	3.4^a^	4.3^b^
Humus (%)	17^a^	22^ab^	40^b^	48^b^
TOC (%)	10^a^	23^ab^	25^b^	31^b^
Total N (%)	0.5^a^	0.8^ab^	1.1^bc^	1.2^c^
Ratio C/N	20^a^	29^c^	23^ab^	25^bc^
P (mg kg^−1^ dry mass)	22^a^	39^ab^	44^ab^	57^b^
K (mg kg^−1^ dry mass)	120^a^	370^b^	347^ab^	416^b^
Mg (mg kg^−1^ dry mass)	201^a^	282^b^	208^ab^	380^b^
Electrolytes (mg KCl kg^−1^ dry mass)	51^a^	184^ab^	364^bc^	448^c^
Autumn				
pH (CaCl_2_)	4.6^c^	4.1^b^	3.4^a^	4.1^b^
Humus (%)	44^a^	57^b^	53^b^	61^b^
TOC (%)	14^a^	32^b^	40^b^	42^b^
Total N (%)	0.6^a^	0.9^a^	1.4^b^	1.4^b^
Ratio C/N	20^a^	29^c^	23^ab^	25^b^
P (mg kg^−1^ dry mass)	27^a^	41^ab^	59^b^	62^b^
K (mg kg^−1^ dry mass)	117^a^	318^ab^	440^b^	528^b^
Mg (mg kg^−1^ dry mass)	237^a^	331^ab^	268^ab^	424^b^
Electrolytes (mg KCl kg^−1^ dry mass)	268^a^	409^ab^	610^b^	657^b^

Different letters in a row denote significant differences (*P* < 0.05) between sites

Continuous monitoring of soil temperature at the studied sites over 1 year showed significantly lower mean annual soil temperatures at the subalpine and alpine sites (R and S; 4.3 and 3.9 °C) than at the submontane and montane sites (M and K; 9.8 and 9.3 °C); comparable trends were observed for minimum and maximum soil temperatures (Table 2). Sites R and S at the higher altitudes were exposed to soil temperatures of or below 0 °C between December and April (i.e. 5 months per year), while the sites at the lower altitudes (M, K) reached subzero temperatures only between December and February or not at all. Maximum soil temperatures were highest in summer and reached ca. 16–20 °C at lower and ca. 12–13 °C at higher altitudes (Siles et al. 2016). The role of microclimate in soil biological processes is rarely considered although it is known to cause temporal variation in microbial structure and activity (Jing et al. 2014).

### Soil sampling and sample preparation

Eight sampling spots distributed uniformly over each site (100 × 100 m) were chosen to cover within-site variability (Siles et al. 2016). Soil samples (ca. 5 kg) were collected from each of these sampling spots from the topsoil (top 10 cm), the number of sampled cores (2–5) depended on the thickness of the topsoil at each site. To determine the effect of season and to take into account the different vegetation periods at the investigated sites, soil samples were collected both in late spring (sites M and K: 24 April 2014; sites R and S: 3 June 2014) and autumn (sites M and K: 15 November 2014; sites R and S: 15 October 2014). Immediately after sampling, soil samples were transported in cooled boxes to the laboratory, sieved (<2 mm) and immediately analysed for culturable microorganisms. Each soil sample was analysed with three replicates.

### Isolation and enumeration of culturable heterotrophic aerobic soil bacteria

For each of the eight soil samples collected per site, 10 g of soil were suspended in 90 mL sodium pyrophosphate solution (0.1 % w/v) and appropriate dilutions were surface spread, in triplicate, onto R2A (Difco) agar plates supplemented with cycloheximide (400 μg mL^−1^) to exclude fungal growth. The plates were wrapped in plastic bags to prevent evaporation and incubated for 28 days at 0 °C (strains isolated at sites M, K, R, S in spring/autumn were designated M0/AM0, K0/AK0, R0/AR0, S0/AS0) and for 7 days at 20 °C (strains isolated in spring and autumn were designated M20/AM20, K20/AK20, R20/AR20, S20/AS20). After incubation, visible colonies were counted and colony forming units (cfu) were calculated on a soil dry mass (dm) basis. For statistical analyses, the mean values of the three replicate plates per soil sample were used. Selected colonies (about 100 per site, season and isolation temperature) were subcultured in the same medium for purification. Purified isolates were stored at −80 °C in R2A broth supplemented with 15 % (w/v) glycerol and preserved at the Institute of Microbiology, University of Innsbruck, Austria.

### Isolation and enumeration of culturable soil yeasts

For each of the eight soil samples collected per site, 10 g of soil were suspended in 90 mL sodium pyrophosphate solution (0.1 % w/v) and appropriate dilutions were surface spread, in triplicate, onto Rose Bengal agar (RB, Difco) and YMA (Difco) agar plates (Nagahama et al. 2001, 2003; Turchetti et al. 2008, 2013), both supplemented with tetracycline (35 μg mL^−1^) to exclude bacterial growth. The plates were incubated for 12 weeks at 4 °C and 3 weeks at 20 °C. Likewise to bacteria, visible colonies grown after incubation were counted and cfu were calculated on a soil dm basis. For statistical analyses, the mean values of the three replicate plates per soil sample were used. Yeast colonies were selected for isolation on the basis of their morphology, taking care to isolate all types occurring at the different incubation temperatures, and frequency. All yeast isolates (about 50 per site, season and isolation temperature) were stored at −80 °C into cryovials containing Protect Microorganism Preservation Beads (Technical Service Consultants Ltd, Lancashire, UK) and preserved in the Industrial Yeasts Collection DBVPG (http://www.dbvpg.unipg.it).

### Phylogenetic analysis of culturable bacteria

Random amplified polymorphic DNA (RAPD) analysis was used as primary fingerprinting method to recognize genomic diversity among the isolates following procedures described before (França et al. 2015). Representative strains of each unique RAPD profile were selected. DNA of representatives of each RAPD group were extracted following the methodology described by Marmur (1963). The 16S rRNA gene was amplified with universal primers 27F and 1525R (Rainey et al. 1996). Amplicons were purified and partially sequenced by Beckman Coulter Genomics (Beckman Coulter Ltd, High Wycombe, UK**)**. Raw 16S rRNA gene sequences were manually inspected using the Sequence Scanner Software V1.0 (Life Technologies Corporation, Carlsbad, CA, USA). SINA stand-alone version 1.2.11 (Pruesse et al. 2012) was used for the automatic alignment of all partial 16S rRNA gene sequences using the Living Tree Project (LTP) release 119 (Yarza et al. 2008) as reference. Aligned sequences were manually improved following the reference alignment in ARBeditor implemented in the ARB software package (Ludwig et al. 2004). Start and end columns of the alignments were trimmed in order to obtain sequences with the same length and information (=750 nt). Representatives of each unique 16S rRNA ribotype were identified using a home-made python script, full sequenced and finally assembled. Operational Taxonomic Units (OTUs) were created based on a near full sequence similarity of 98.7 % using the script pick_otus.py implemented in the QIIME software package (Caporaso et al. 2010) applying the UCLUST clustering method (Edgar 2010). Traditionally, 97 % 16S rRNA gene sequence similarity has been generally accepted as the threshold for the delimitation at the species level (Stackebrandt and Goebel 1994). However, given constants developments in taxonomic studies, the species threshold was suggested to be raised to 98.7–99.0 % (Stackebrandt and Ebers 2006). We opted to use the level of 98.7 % to define taxa even though this cut-off threshold may, in some taxa, underestimate the number of species due to the low phylogenetic resolution of the 16S rRNA gene (Rosselló-Móra 2012). Aligned near full length sequences of representatives for each OTU were inserted into the LTPs119 tree using the ARB-parsimony tool implemented in the ARB software and closely related reference sequences were selected. A de novo phylogenetic reconstruction of isolates sequences with the reference sequences was calculated using the RAxML algorithm with the GTRGAMMA model (Stamatakis 2006), with a 40 % maximum frequency filter to remove noise and preserve positional orthology. OTUs with identity values >98.7 % and with type strain reference sequences were considered to belong to the same species. For identity values >94.5 % with the closest relative type strain 16S rRNA gene sequence, OTUs were considered to be of the same genus (Yarza et al. 2014).

### Identification and phylogenetic analysis of culturable yeasts

Total genomic DNA was extracted according to the study by Turchetti et al. (2008) with some modifications. After checking the sufficient amount of DNA, the synthetic oligonucleotides (GTG)5 and M13 (Sigma-Aldrich) were used as single primers for MSP-PCR fingerprinting (Libkind et al. 2003). DNA was amplified using a T personal Combi Thermal Cycler (Biometra^®^ GmbH). Amplification products were separated by electrophoresis (Sampaio et al. 2001; Gadanho and Sampaio 2002); strains exhibiting identical DNA banding patterns were grouped and considered to belong to the same species.

Isolates were subjected to sequencing of the D1/D2 domain of 26S rRNA gene. DNA was first amplified using the primers RLR3R (5′-GGTCCGTGTTTCAAGAC-3′) and V9 (5′-TGCGTTGATTACGTCCCTGC-3′; Sigma-Aldrich). A 600–650-bp region was sequenced by the forward primer NL1 (5′-GCATATCAATAAGCGGAGGAAAAG-3′) and the reverse primer NL4 (5′-GGTCCGTGTTTCAAGACGG-3′; Sigma-Aldrich). Sequencing of Internal Transcribed Spacers (ITS1&2) including the 5.8S rRNA gene region was performed for all those strains exhibiting ambiguous results of D1/D2 sequences. A 600–650-bp region was sequenced by the forward primer ITS1 (5′-TCCGTAGGTGAACCTGCGG-3′) and the reverse primer ITS 4 (5′-TCCTCCGCTTATTGATATGC-3′; Sigma-Aldrich). All sequences were determined by a commercial sequencing facility (Macrogen). Alignments were made using Vector NTI Suite 8 Contig Express (Informax, Invitrogen). In both cases, strains were identified by comparing the sequences obtained with the GenBank database (BLASTN freeware from http://www.ncbi.nlm.nih.gov/BLAST). Phylogenetic analysis was performed using molecular evolutionary genetics analysis (MEGA) software 6 version 4.1 (Tamura et al. 2007) using neighbor-joining analysis. Bootstrap analysis (1000 replicates) was performed using a full heuristic search.

### Statistical analysis

The mean values of replicate determinations of cfu and soil physicochemical parameters were used for statistical calculations (Statistica, version 9.0). Normal distribution was tested with the Kolmogorov–Smirnov test. ANOVA was applied to determine whether the sampling season and/or the sampling site (altitude) had a significant (*P* < 0.05) effect on soil bacterial and yeast numbers and soil properties. Correlation analyses between bacterial and yeast numbers, biodiversity indices and soil physicochemical parameters were performed with the Pearson product–moment correlation test.

The PAST software v1.82 b (Hammer et al. 2001) was used to compute the statistical diversity indexes in the datasets. Shannon–Wiener, Dominance-D and Pielou’s evenness indexes were computed with a bootstrap percentile type procedure (9999 iterations). A 95 % confidence interval was calculated and mean values were shown. Pielou’s evenness index, species richness, species frequency, frequency of rare species and number of singletons were calculated for yeasts (Yurkov et al. 2016). Individual rarefaction curves were calculated using a log Gamma function for computing combinatorial terms. Calculations were made for Good’s coverage: *C* = 1 − (ni*/*nt), where ni is the number of bacterial OTUs or yeast species observed exactly once and nt is the total number of OTUs or species, as reported by Li et al. (2012).

Principal component analysis (PCA) of the data was performed using CANOCO version 4.5 (Microcomputer 4.5 Power). In order to reduce the input data size and to draw the major changes between sites, we decided to group the isolates in bacterial OTUs and yeast species with the less stringent sequence identity cut-off value of 97 %. Scaling was focused on inter-sample distances and bacterial OTU and yeast species scores divided by standard deviation. Both bacterial OTUs and yeast species (%) data were not transformed and results were centred by species. To avoid the overcrowding of the graphs, only species having a contribution to the analysis higher than 25 % were represented. PCA graphs were constructed (1) to infer differences in strain collections recovered at the two isolation temperatures, and (2) to detect correlations between altitude and season on the total culturable bacterial and yeast diversity per site.

### Accession numbers

The 16S rRNA gene sequences of representatives of each bacterial OTU were deposited in GenBank under the accession numbers KP899142 to KP899253. Likewise, the 26S rRNA gene and ITS1&2 sequences of representatives of each yeast species were deposited in GenBank under the accession numbers KU745308 to KU745323, KU745325 to KU745327, KU745329 to KU745330, KU745332 to KU745381.

## Results

### Bacterial numbers

**Fig. 1:**
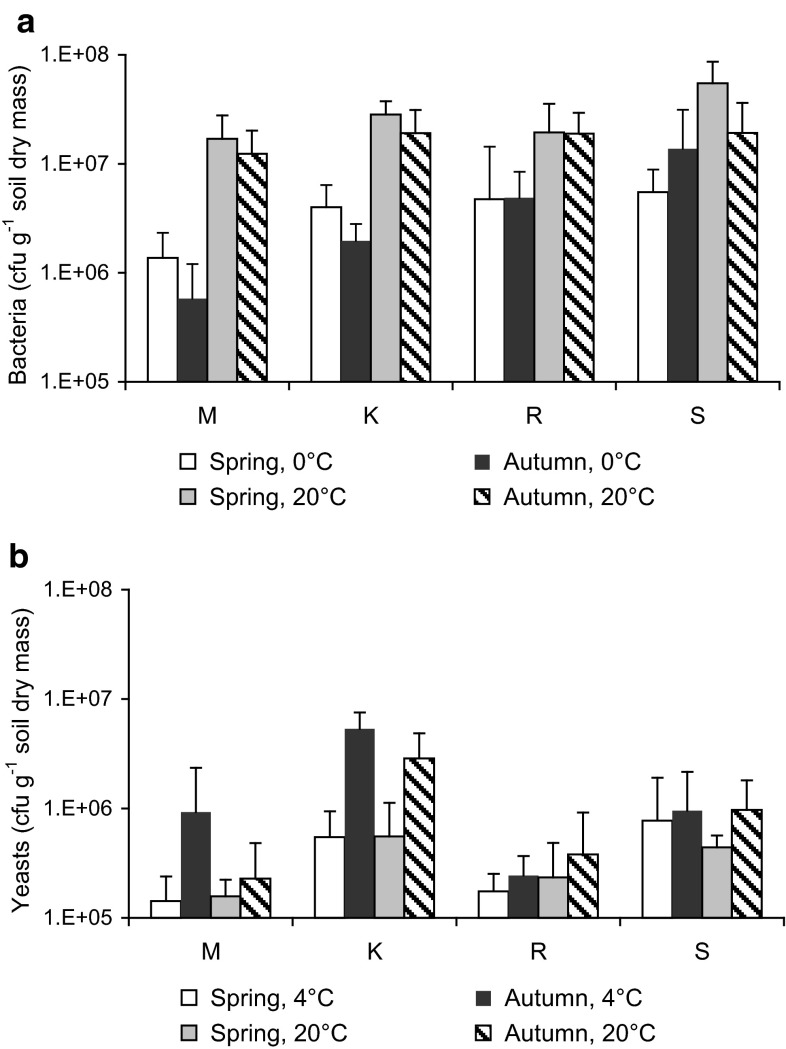
Numbers of colony forming units (cfu) of culturable heterotrophic bacteria at 0 and 20 °C (**a**) and of culturable yeasts at 4 and 20 °C (**b**) at the at the investigated sites in spring and autumn. Values are the mean of three independent replicates

For culturable bacteria isolated at 0 and 20 °C we use the terms psychrophilic and mesophilic, respectively. The term “psychrophilic” is used as a general term to describe a microorganism that grows well in a cold environment. The use of growth rates to define the optimum growth temperature as described by Morita (1975) has been shown to be ambiguous and inappropriate (Feller and Gerday 2003; Cavicchioli 2006; Margesin 2009), since the apparent “optimal” temperature for growth gives no indication about the microbial ability to thrive well at low temperatures. Abundance (numbers) of culturable soil bacteria at the four sites, isolated at 20 °C, was (1.7–5.5) × 10^7^ and (1.2–1.9) × 10^7^ cfu g^−1^ soil dm in spring and in autumn, respectively. Numbers of bacteria at 0 °C were generally (at each of the sites, except at the alpine site S in autumn) significantly (*P* < 0.05–0.001) lower than those recovered at 20 °C and consisted of (1.4–5.5) × 10^6^ and (0.6–14) × 10^6^ cfu g^−1^ soil dm in spring and in autumn, respectively (Fig. 1a). The major differences were observed in autumn for the sites at the lowest (submontane site M) and at the highest (alpine site S) altitude. At the submontane site M the differences between bacterial counts at 0 and 20 °C were significantly higher in autumn than in spring (due to a decrease of numbers at 0 °C), while the opposite was observed for the alpine site S (due to an increase of number at 0 °C). Altitude influenced generally soil bacterial numbers in both seasons significantly (*P* < 0.05), however, this effect was much more pronounced in autumn than in spring. Abundance of psychrophiles was not significantly different at any of the four sites in spring, while a significant increase of psychrophiles with altitude (from 5.8 × 10^5^ cfu g^−1^ soil dm at the submontane site M to 1.4 × 10^7^ cfu g^−1^ dm soil at the alpine site S) could be recognized for autumn.

This was reflected by the ratios between the mesophilic and psychrophilic fraction of the culturable bacterial community which were significantly influenced by altitude in autumn but not in spring. In autumn, a constant increase in the cfu ratio 0 °C/20 °C (0.05, 0.10, 0.26, and 0.72 at sites M, K, R, S, respectively) and accordingly a constant decrease in the cfu ratio 20 °C/0 °C occurred with altitude (21, 9.8, 3.9 and 1.4 at sites M, K, R, S, respectively). In addition, the relative fraction of mesophiles was significantly (*P* < 0.001) higher at the site at the lowest altitude (submontane site M) compared to the other three sites, while the relative fraction of psychrophiles was significantly (*P* < 0.001) higher at the site at the highest altitude (alpine site S) than at the other sites.

### Yeasts numbers

Although yeasts cannot be effectively split between psychrophiles and mesophiles because most of known species isolated from soil grow over a wide range of temperatures, the terms psychrophilic and mesophilic have often been used to categorize yeasts isolated at 4 and 20 °C, respectively (Turchetti et al. 2008, 2013). In contrast to bacterial counts, no clear trends were observed in dependence of the temperature of isolation, season and/or altitude. Overall, yeast abundance (numbers) at 4 °C did not significantly (*P* < 0.05) differ from those observed at 20 °C, except at the submontane site M in autumn. Psychrophilic yeast numbers were (1.4–7.7) × 10^5^ cfu g^−1^ soil dm in spring and ranged from 2.4 × 10^5^ to 5.3 × 10^6^ in autumn; mesophilic yeast numbers were from (1.5–5.5) × 10^5^ in spring and ranged from 2.3 × 10^5^ to 2.9 × 10^6^ in autumn (Fig. 1b). Significant (*P* < 0.05) seasonal differences with higher yeast numbers in autumn than in spring were only found at the montane site K (Fig. 1b).

Like for bacteria, the ratios between the mesophilic and psychrophilic fraction of the culturable yeast community were influenced by altitude in autumn, but not in spring. In autumn, a constant decrease in the cfu ratio 4 °C/20 °C (4.1, 1.9, 0.6, and 1.0 at the submontane, montane, subalpine and alpine sites M, K, R, S, respectively) and accordingly a constant increase in the cfu ratio 20 °C/4 °C occurred with altitude up to site R. In comparison, bacteria showed an opposite pattern with a constant increase in psychrophiles over the whole altitudinal gradient.

### Effect of isolation temperature on the culturable bacterial diversity

A total number of 1194 bacterial strains was isolated in spring (609 strain) and autumn (585 strains) from soils of the four different sites. Colonies recovered from initial isolations incubated at 0 and 20 °C were screened using RAPD technique to reduce clonal redundancies among the strains isolated at the two temperatures. The 16S rRNA genes of representatives of all generated clusters were partially sequenced and unique ribotypes were near full sequenced. OTUs were created using a 16S rRNA gene sequence similarity of 98.7 %.

Good’s coverage values for samples isolated at 0 °C (87–99 %) indicated a sufficient sampling effort to cover the diversity of the culturable bacteria under the conditions examined (Table 3). Lower coverage values (66–83 %) were obtained for isolations performed at 20 °C. Rarefaction calculations also showed that an increase of the surveying effort would be necessary to cover the full extent of the culturable diversity at 20 °C by the plotted curves failing a saturation phase (Fig. S1). The lowest dominance-D (DD), highest Shannon–Wiener (SW), highest evenness (EN) indexes and highest numbers of singletons (SI) were obtained for isolations performed at 20 °C (DD 0.04–0.09; SW 2.8–3.3; EN 0.53–0.78; SI 1–11) as a reflection of a higher culturable bacterial diversity at this temperature (Table 3). For isolations at 0 °C, the values were (DD) 0.13–0.23, (SW) 1.8–2.5, (EN) 0.53–0.78 and (SI) 1–11 demonstrating that fewer taxa dominated the culturable bacterial community at the low temperature.

**Table 3:** Diversity indexes obtained for bacterial diversity at 0 and 20 °C and for yeast diversity at 4 and 20 °C at the four sites in spring and autumn

Isolation temperature	Bacteria												
	0 °C								20 °C				
Season	Spring				Autumn				Spring				
Site	M submontane	K montane	R subalpine	S alpine	M submontane	K montane	R subalpine	S alpine	M submontane	K montane	R subalpine	S alpine	M submontane
Strain designation	M0	K0	R0	S0	AM0	AK0	AR0	AS0	M20	K20	R20	S20	AM20
Number of OTUs	21	21	10	14	9	10	10	9	34	32	26	35	34
Number of isolates	82	101	69	94	63	79	86	83	75	60	61	67	62
Dominance-D index	0.13	0.15	0.21	0.18	0.23	0.18	0.22	0.16	0.05	0.04	0.06	0.06	0.05
Shannon-Wiener index	2.5	2.4	1.8	2.1	1.7	1.9	1.8	1.9	3.2	3.3	3.0	3.2	3.3
Good’s Coverage (%)	86.6	93.1	94.2	96.8	96.8	97.5	98.8	98.8	77.3	70	82	65.7	67.7
Evenness index	0.56	0.53	0.61	0.56	0.61	0.66	0.60	0.78	0.75	0.84	0.78	0.73	0.78
Singletons (N)	11	7	4	3	2	2	1	1	17	18	11	23	20

Isolation temperature	Yeasts												
	4 °C								20 °C				
Season	Spring				Autumn				Spring				
Site	M submontane	K montane	R subalpine	S alpine	M submontane	K montane	R subalpine	S alpine	M submontane	K montane	R subalpine	S alpine	M submontane
Strain designation	M4	K4	R4	S4	AM4	AK4	AR4	AS4	M20	K20	R20	S20	AM20
Species richness (N)	14	8	7	9	6	8	11	12	7	5	6	8	4
Number of isolates	99	104	50	69	40	62	47	56	36	33	18	43	6
Dominance-D index	0.27	0.40	0.45	0.41	0.56	0.40	0.26	0.30	0.25	0.27	0.22	0.36	0.33
Shannon–Wiener index	1.7	1.2	1.1	1.3	0.9	1.2	1.7	1.6	1.6	1.4	1.6	1.3	1.2
Good’s Coverage (%)	85.9	92.3	86.0	86.9	85.0	87.1	76.6	78.6	80.6	84.8	66.7	81.4	33.3
Evenness index	0.64	0.58	0.57	0.59	0.5	0.58	0.74	0.67	0.82	0.87	0.89	0.62	0.86
Frequent species (%, >10 %)	14	25	28	22	33	25	20	18	43	60	66	25	100
Rare species (%, <1 %)	0	12	0	0	0	0	0	0	0	0	0	0	0
Singletons (N)	7	1	3	4	4	4	4	7	2	0	2	5	3

Based on the phylogenetic inference, we recovered 112 OTUs belonging to the phyla *Proteobacteria (*classes *Alpha*-, *Beta*- and *Gammaproteobacteria)*, *Bacteroidetes* (*Spingobacteriia*, *Cytophagia* and *Flavobacteriia*), *Actinobacteria* (*Actinobacteria*) and *Firmicutes* (*Bacilli*) (Fig. S2). All phyla were present at a comparable relative incidence among strains recovered at 0 °C (psychrophiles) and 20 °C (mesophiles). *Gammaproteobacteria* and *Betaproteobacteria* dominated both psychrophiles and mesophiles. While *Gammaproteobacteria* were more present among psychrophiles (47–91 % of the relative fraction), *Betaproteobacteria* were found to a higher extent among mesophiles (23–58 %) (Fig. S3).

All bacterial OTUs affiliated with known genera considering the 16S rRNA minimal similarity threshold of 94.5 %, but 57 of 112 OTUs could constitute potential novel species using the stringent similarity value of 98.7 % (Table S1). Using a less conservative threshold, 33 of these OTUs shared 16S rRNA gene identities ≤98.0 % with their closest relative type strains. The majority (41 of 57 OTUs) of the potential novel species were present among mesophiles.

**Fig. 2:**
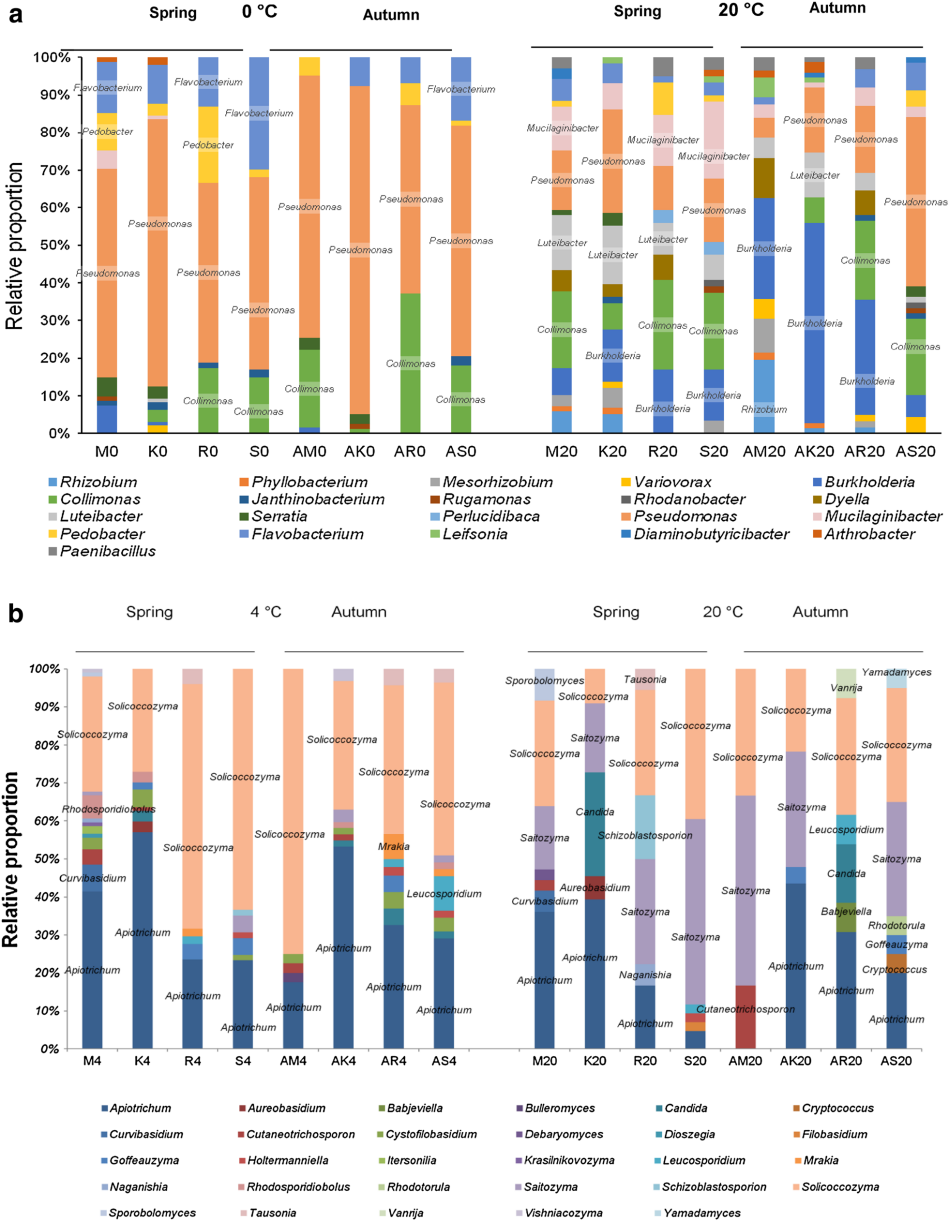
Relative proportions of the different bacterial genera isolated at 0 and 20 °C (**a**) and of the different yeast genera isolated at 4 and 20 °C (**b**) in spring and autumn. Only genera with more than three strains are shown. Dominant taxa present in all data subsets (at each site, in each season and at each isolation temperature) are boxed

Taxonomic assignment indicated that representatives of the OTUs were affiliated with 41 different genera. In general, *Pseudomonas* was the most represented genus and accounted for 504 strains, representing 42 % of the total number of isolates (Fig. 2a; Table S1). Nine OTUs could be affiliated within *Pseudomonas* (OTU 51–59). OTU 53, with 182 strains, was the most present group and was related to *P. lini* (99.4 % identity). OTU 53 was present in all of the sites (except at 0 °C at submontane site M in autumn; AM0) and was especially found among psychrophiles (11–33 %). Remaining phylogenetically close relatives were *P. constantinii*, *P. psychrophila*, *P. japonica*, *P. lutea*, and *P. jessenii* but 4 of the 9 OTUs showed only a similarity value of <98.7 % with valid type strains present in the LTP database. The genus *Collimonas* was the second most represented genus and accounted for 166 strains, representing 13 % of the total number of isolates. This genus harboured 3 OTUs, one of which was a putative novel species (OTU 32, one strain, 97.5 % identity with *C. pratensis*). OTUs 30 and 31 were related to *C. fungi*-*vorans* and *C. pratensis* (>99.8 % identity).

There was phylogenetic overlap in some major populations isolated from most of all sites in both seasons; some taxa were mainly isolated at 0 °C [e.g. *Flavobacterium* (OTU 82)], some mainly at 20 °C [e.g. *Burkholderia* (OTUs 17 and 25) and *Luteibacter* (OTU 44)] and some taxa were isolated at both temperatures [e.g. *Collimonas* (OTU 31), *Pseudomonas* (OTUs 53, 54 58)]. The strains recovered at 0 and 20 °C drew different number of OTUs; 76 of 112 OTUs were exclusive of mesophiles, 7 OTUs were recovered only at 0 °C and 29 OTUs were detected at both temperatures. The most present OTUs were recovered at both isolation temperatures (e.g. OTUs 31, 52–54, 58, and 59). OTUs that represented potential novel species were always present in low numbers and restricted to one or two sites.

The relative proportions of the genera recovered at 0 and 20 °C showed a different composition of the culturable heterotrophic community at these two temperatures (Fig. 2a). At 0 °C, the dominant strains belonged mainly to *Pseudomonas* spp. (mainly OTUs 51, 53, 54 and 58) but also to *Collimonas* spp. (OTU 31) and to *Flavobacterium* spp. (mainly OTUs 82 and 84). As indicated above, the diversity of culturable bacteria at genus level for isolations at 20 °C was always higher than those performed at 0 °C, supporting information of alpha diversity indices. Culturable diversity at all four sites at 20 °C was distributed evenly by a more diverse number of genera but consisting mainly of *Mucilaginibacter* spp. (mainly OTUs 70, 71), *Pseudomonas* spp. (OTUs 53, 54), *Luteibacter* spp. (OTUs 43, 44), *Collimonas* spp. (OTUs 30, 31), *Burkholderia* spp. (mainly OTUs 17, 21 and 25) and *Rhizobium* spp. (OTU 1) (Fig. 2a; Table S1).

Most members of the classes *Bacilli* (genera *Paenibacillus*, *Psychrobacillus* and *Bacillus*) and *Actinobacteria* (genera *Marmoricola*, *Leifsonia*, *Plantibacter* among others) and all members of the *Alphaproteobacteria* (genera *Rhizobium*, *Phyllobacterium*, *Mesorhizobium* among others) were minor populations but exclusively recovered at 20 °C (Table S1).

**Fig. 3:**
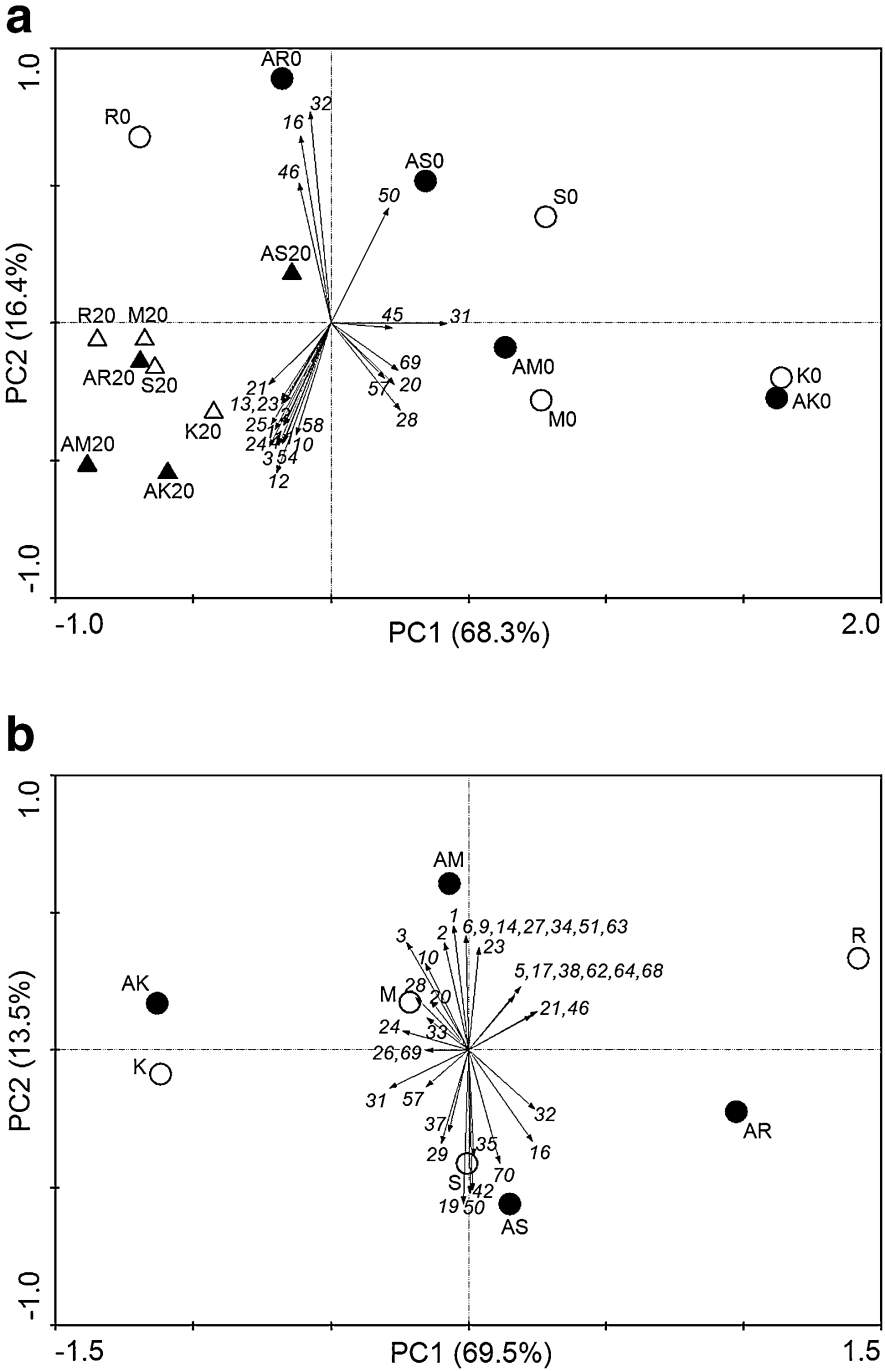
Principal component analysis as a function of the relative abundance (%) of bacterial OTU. **a** Strains per site isolated at 0 °C (*circle*) and 20 °C (*triangle*) in spring (*white filled*) and autumn (*black filled*). **b** Total diversity per site in spring (*white filled*) and autumn (*black filled*). *Numbers* refer to OTU IDs (Table S1)

For principal component analysis (PCA) we decided to reduce the 16S rRNA similarity threshold value for OTU definition to 97 % in order to decrease the dataset size and to detect the overall patterns of taxa variation across the sites. Strains isolated at 20 °C formed a coherent cluster in the lower left quadrant of the PCA plot, showing similarity in taxa composition and incidence across the datasets that were different from the datasets of psychrophiles (Fig. 3a). On the other hand, psychrophiles were separated mainly along PC2 axis, which, however, only explained 13.5 % of total variance. The cluster created by strains from the submontane site M and the montane site K collected in both seasons was distinct from the cluster created by strains from the subalpine site R and the alpine site S, which demonstrates differences in bacterial community composition at lower and higher altitudes. The separation of strains from submontane and montane sites (M and K) was influenced by the OTUs 20, 28, 31, 45, 57 and 69, while the distinction of strains from subalpine and alpine sites (R and S) was derived mainly from OTUs 16, 19, 32, 46 and 50.

### Effect of isolation temperature on the culturable yeast diversity

Seven hundred and nineteen yeast strains were isolated in spring (452 strains) and autumn (267) from soils sampled at the four sites. Due to lower numbers of morphotypes found on agar plates in autumn than in spring, fewer yeast strains were recovered in autumn (when yeast numbers tended to be higher than in spring, however, without any statistical significance, Fig. 1b) Likewise to bacteria, yeast colonies recovered from initial isolations incubated at 4 and 20 °C were screened using MSP-PCR fingerprinting technique to reduce the possible clonal redundancy among the isolates. Strains were identified at the species level using D1/D2 of the 26S rRNA gene and internal transcribed spacers (ITS1&2) including the 5.8S rRNA gene sequences similarity as high as 98 %.

Good’s coverage values were high for isolations at 4 °C at all four sites in spring (86–92 %), at 4 °C at the sites M, K, S (submontane, montane and alpine, respectively) in autumn (79–87 %), and for isolations at 20 °C at the same sites in spring (81–85 %) and at site K in autumn (83 %). Thus, in these cases the sampling effort was able to cover a high degree of diversity of the culturable yeasts under the examined conditions. In contrast, lower coverage values were obtained for isolations performed at the subalpine site R at 4 °C in autumn (77 %) and at 20 °C in spring (67 %), and for isolations at 20 °C at the sites M, R, S in autumn (33–60 %) (Table 3). This suggested that an increase of the surveying effort would be necessary to cover a higher degree of the culturable yeast diversity at those sites. Rarefaction calculations showed that the culturable yeast diversity both at 4 and 20 °C apparently reached a saturation phase (Fig. S1). DD and SW indexes exhibited similar values for isolations performed at 4 °C (DD 0.27–0.45 and 0.27–0.56; SW 1.1–1.7 and 0.9–1.6; in spring and autumn, respectively) and 20 °C (DD 0.22–0.36 and 0.20–0.33; SW 1.3–1.6 and 1.2–1.8; in spring and autumn, respectively) (Table 3).

The proportion of the different species in the yeast community, as evaluated by calculating EN, exhibited different distribution patterns in dependence of the isolation temperature: isolations at 4 °C at all four sites showed lower values (0.50–0.74) than those at 20 °C (0.82–0.89), with the sole exception of the alpine site S in spring (0.62) (Table 3). This trend was confirmed by the percentage of the most frequent species (>10 % of isolates), which was lower at 4 °C (14–33 %) than at 20 °C (37–100 %), Also in this case, isolations at 20 °C at site S in spring exhibited a contrasting value (25 %). These data demonstrated that the temperature of isolation significantly affected yeast species evenness: few yeast species dominated the culturable diversity at 4 °C (Table 3). Overall, with the sole exception of isolations at 4 °C at the montane site K in spring, no rare species (<1 % of isolates) were found. In contrast, a few singletons were generally found at all four sites (Table 3). Based on the phylogenetic inference, we recovered 36 species belonging to the classes *Dothideomycetes* (order *Dothideales*), *Saccharomycetes (Saccharomycetales)* (Fig. S4), *Tremellomycetes* (*Filobasidiales*, *Holtermanniales*, *Tremellales*, *Trichosporonales* and *Cystofilobasidiales*), and *Mycrobotryomycetes* (*Sporidiobolales*, *Leucosporidiales*, *Kriegeriales*, and *Incertae sedis*) (Fig. S5). Taxonomic assignment of isolates indicated that the 36 species were affiliated to 29 different genera: 4 belonging to Ascomycota, 24 to Basidiomycota and one to yeast-like dimorphic fungi (*Aureobasidium*) (Fig. 2b).

Likewise to bacterial populations, a phylogenetic overlap was observed in some major yeast communities isolated from most of the sites in both seasons. Overall, basidiomycetous yeasts dominated both psychrophiles and mesophiles, while ascomycetes represented only a tiny fraction of yeast culturable diversity (1–8 %). The sole exception of this general trend were the species *Candida santamariae*, *Candida oregonensis* and *Schizoblastosporion skarkeyi*-*henricii*: strains belonging to the first two species were detected in percentages higher than 10 % at 20 °C at the montane site K in spring and at the subalpine site R in autumn (27 and 15 %, respectively), while 17 % of strains isolated at 20 °C at site R in spring belonged to *S. skarkeyi*-*henricii* (Fig. S6). The basidiomycetous species *Apiotrichum porosum* and *Solicoccozyma terricola* covered 15–55 and 25–75 % of the relative fraction, respectively, of the yeast diversity found at 4 °C and a consistent part (5–40 and 5–45 %, respectively) of that at 20 °C. On the contrary, *Saitozyma podzolica* was the most abundant species at 20 °C at the sites R and S (subalpine and alpine) in spring (28–49 %) and at the sites M and S (submontane and alpine) in autumn (50–30 %) (Fig. S6). Some species were exclusively found at 4 °C: among them *Bullera alba*, *Cystofilobasidium capitatum*, *Dioszegia hungarica*, *Itersonilia pannonica*, *Mrakia gelida*, *Rhodosporidiobolus colostri* and *Vishniacozyma victoriae*. On the contrary, the species *Babjeviella inositovora*, *Debaryomyces hansenii*, *Vanrija musci* and *Yamadamyces rosulatus* were isolated exclusively at 20 °C (Fig. S6).

A handful of yeast strains (less than 1 % of isolates) exhibited a homology below 98 % of D1/D2 and ITS1&2 sequences with those of the closest species. They could constitute four potentially novel basidiomycetous species (about 10 % of the total observed species) presumably belonging to the genera *Piskurozyma*, *Filobasidium*, *Krasilnikovozyma* and *Rhodototula* (Fig. S5).

**Fig. 4:**
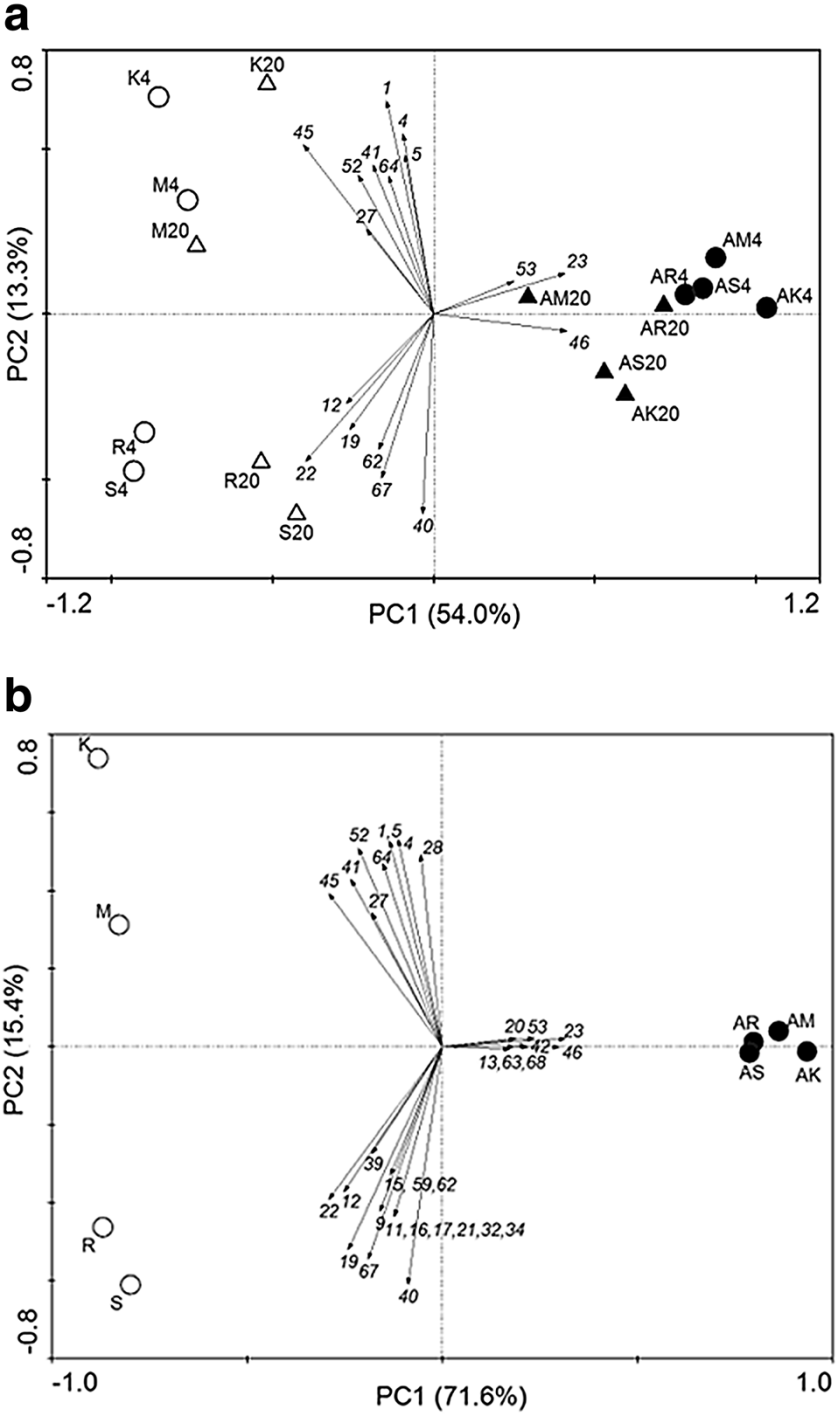
Principal component analysis as a function of the abundance of the yeast population at the species level. **a** Strains per site isolated at 4 °C (*circle*) and 20 °C (*triangle*) in spring (*white filled*) and autumn (*black filled*). **b** Total diversity per site in spring (*white filled*) and autumn (*black filled*). *Numbers* refer to species IDs (Table S2)

PCA was performed at the species level in order to detect the overall patterns of yeast taxa variation across the sites. In contrast to bacteria, yeasts isolated at 4 and 20 °C did not form well defined clusters in the PCA plot (Fig. 4a).

### Effect of altitude and season on the culturable bacterial diversity

There was no significant (*P* > 0.05) correlation between DD and SW indexes related to the altitudinal or seasonal effect. Moreover, it was not possible do discern a clear trend in the culturable bacterial diversity that could be related to altitude or season (Table 3).

PCA performed on the total bacterial diversity (without differentiation between psychrophiles and mesophiles) recovered at each site (Fig. 3b) showed that the total sample variation was mainly explained by the first principal component (69.5 %), the second principal component only explained 13.5 % of the variation. The montane and subalpine sites K and R were highly separated along the PC1 axis while the submontane and alpine sites M and S were distributed along PC2. Data points corresponding to each site sampled in both seasons tended to create independent clusters indicating a stable biodiversity that could be site specific.

### Effect of altitude and season on the culturable yeast diversity

SW index, species richness, species frequency, frequency of rare species and number of singletons indicated apparently no significant (*P* > 0.05) correlation for the whole yeast diversity in relation to the altitudinal or seasonal effect. On the other hand, DD index exhibited a decreasing trend with altitude in autumn (Table 3). In addition, PCA performed at the species level demonstrated season-dependent differences in yeast community structure: yeasts isolated in spring showed a clear separation (left quadrant of the PCA plot) from those isolated in autumn (Fig. 4).

### Correlations

Correlation analyses between microbial numbers and soil physicochemical properties revealed a significantly (*P* < .0.05) positive impact of soil nutrients (TOC, total N, P, K, Mg, electrolytes) on numbers of psychrophilic bacteria, but not on numbers of mesophilic bacteria. The bacterial cfu ratio 20 °C/0 °C correlated significantly with soil pH and soil nutrients (TOC, total N, P, K). A completely different pattern was observed with yeasts. No significant correlations of soil nutrients or soil pH with numbers of psychrophiles, mesophiles or ratios were found. However, there was a significantly negative correlation between the ratios of mesophilic and psychrophilic bacterial and yeast populations.

A similar result was obtained with correlation analyses between soil physicochemical properties and biodiversity indices. DD index of psychrophilic and mesophilic bacteria correlated significantly positively with soil humus and TOC contents and with electrolytes, A significant positive correlation was also found with bacterial SW index and contents of humus (psychrophilic bacteria) or TOC and electrolytes (mesophilic bacteria). In contrast, no significant correlations were detectable between yeasts diversity indices and soil nutrients. Soil pH correlated neither significantly with bacterial nor with yeast diversity indices.

## Discussion

Forests are expected to face significant pressures in the future to overcome the deleterious effects of global warming due to climate change. However, knowledge on the effect of changed climate conditions on soil microbiota in Alpine forest ecosystems is still insufficient. Therefore, we surveyed soil culturable bacterial and yeast communities in spring and autumn along an altitude gradient that covered submontane, montane, subalpine and alpine vegetation belts.

It has become canonical that culturable microbial diversity does not appear to represent microorganisms detected with molecular surveys. However, it is worth of note that studies on bacteria using both culture-dependent and culture-independent methods have shown that these datasets had very few taxa in common, which indicates that an important fraction of microbial diversity would have been disregarded if only the culture-independent dataset had been analyzed (Donachie et al. 2007). However, there is hardly any study comparing culture-dependent and culture-independent surveys of yeasts. Also, cultivation-based methods might be more sensitive to retrieve changes of the physiologic and metabolic state of the community due to environmental fluctuations since the culturable fraction of the community might react more rapidly to changes in biotic and abiotic factors than genomic surveys that mainly target DNA fragments derived from viable and non-viable organisms (Smit et al. 2001).

The data obtained in our study demonstrate lower abundance of psychrophilic bacteria compared to mesophilic ones, independent of the altitude. The alpine site S, however, had comparable (not significantly different) numbers of psychrophiles and mesophiles in autumn. In this season, the relative fraction of psychrophiles among the culturable bacterial community increased with altitude, while those of mesophiles remained almost stable. This corroborates results from our previous studies on microbial communities along an altitude gradient in the Austrian Central Alps (Margesin et al. 2009). In the present study, a significant increase in the relative amount of psychrophilic bacteria with altitude was not detectable in spring, which indicates that cold-adaptation might be more pronounced in autumn than in spring, possibly as a result of the lower soil temperatures in autumn than in spring. In contrast to bacteria, no clear trends in dependence of the temperature of isolation, season and/or altitude were detected for viable yeast cell numbers, with the sole exception at submontane and montane sites in autumn where a few significant differences were found. Significant differences between bacteria and yeasts were also found for correlations with cfu and soil physicochemical parameters. Soil nutrients had a significantly positive effect on the abundance of bacterial psychrophiles (however, not on mesophiles), which corroborates results obtained with non-culture-based techniques from our previous studies (Siles and Margesin 2016; Siles et al. 2016). The data of the present study demonstrate that the impact of nutrients might be much more pronounced on psychrophilic than on mesophilic bacteria, which has not yet been described before. In contrast, no correlation was detectable between soil properties and yeast abundance. The different distribution patterns of bacteria and yeasts might be explained by the influence of factors not tested in this study on the heterotrophic metabolism of both mesophilic and psychrophilic yeasts, such as the effective availability of nutrients and growth factors in soil, which could be affected by seasonal changes of temperature and precipitation. Our previous study on the influence of abiotic variables on culturable yeast diversity in two distinct Alpine ecosystems demonstrated correlations between yeast abundance and some abiotic factors (i.e. pH and organic nutrients); the abundance of culturable yeasts increased from oligotrophic to eutrophic conditions and was particularly correlated with the content of organic phosphorus (Turchetti et al. 2013). Besides, the significantly negative correlation between the ratios of mesophilic and psychrophilic bacterial and yeast populations could be justified by postulating a competition for organic nutrients between bacteria and yeasts. Trophic competition for nutrients such as carbon, nitrogen or iron between bacteria and fungi (including yeasts) in natural environments has been recently reviewed (Frey-Klett et al. 2011).

The phylogenetic analysis of 1194 isolated bacterial strains rendered a total of 112 different OTUs; temperature was a significant predictor of bacterial community composition across seasons and altitude. Major differences in biodiversity of psychrophilic and mesophilic strains were recognized. The bacterial community isolated at 0 °C was dominated at all sites, i.e. independent of altitude, and in both seasons by a few selected taxa, which points to a low diversity among psychrophiles. The diversity of bacteria isolated at 20 °C at the genus level was always, regardless of altitude or season, higher than that of psychrophiles. More than 50 % of the 112 OTUs could be regarded as potential novel bacterial species according to their phylogenetic affiliation and sequence similarity with the closest related type strain. This remarkably high amount of yet undescribed bacterial taxa points to a large reservoir of unexplored members of microbial communities at the studied forest sites. Further polyphasic characterization is needed to verify the phylogenetic assignment described in this study.

The phylogenetic analysis of 719 yeast isolates rendered a total of 36 different species, of which a few species were numerically abundant. Yeast community structures at the four sites investigated in this study revealed a lower species richness, than those reported by other studies on yeast communities in soils of beech forests, Mediterranean forests, woodlands and scrub biomes (Yurkov et al. 2011; Yurkov et al. 2016). This was also confirmed by the diversity indexes, which indicated that the temperature of isolation affected yeast culturable evenness significantly, since only a few yeast species dominated the culturable diversity at low temperature (Table 3).

There was phylogenetic overlap in some major bacterial and yeast populations isolated from most of all sites in both seasons. Taxa found at both isolation temperatures could be members of a stable microbial population across altitude and season. However, overlapping taxa could be due to the recognized limitations of culture-dependent methods, which could lead to the selection of the same taxa that were able to growth on agar plates at both isolation temperatures. Further studies on carbon source utilization patterns of phylogenetically close strains isolated from the studied sites should reveal the phenotypes adapted to site specific conditions. This could give further insights into the diversity and activity of both prokaryotic and eukaryotic culturable microbiota.

The most frequent bacterial genus was *Pseudomonas*, which has been recursively isolated and reported to be a major culturable genus in soil, including Alpine forest soils (Männistö and Häggblom 2006). In our study, most pseudomonads were psychrophilic (isolated at 0 °C) and were clustered into 10 OTUs (the exception was OTU 56 closely related to *P. japonica*, *n* = 1). This could indicate a high phylogenetic and metabolic diversity among *Pseudomonas* strains as an adaptive response to the myriad of niches with pockets-specific carbon substrates and conditions found in Alpine environments (Meyer et al. 2004). In our study, the most present species among *Pseudomonas* was *P. lini.* Representatives of this species were found in rhizospheric soil (Delorme et al. 2002) and in Antarctica (Arenas et al. 2014). Other frequent genera included *Collimonas*, *Burkholderia*, *Flavobacterium*, *Mucilaginibacter*, *Luteibacter* and *Pedobacter.* These cosmopolitan populations are often found in cultivation studies from soil environments. The genera dominating the psychrophilic population (*Pseudomonas*, *Collimonas* and *Flavobacterium*) are known to prevail in cold soils (Männistö and Häggblom 2006; Zumsteg et al. 2013). A very low proportion of Gram-positive bacteria (3.5 %) was generally recovered. Previous studies indicated that plant roots have a selective effect towards *Proteobacteria*, mainly *Gammaproteobacteria*, to the detriment of Gram-positive bacteria and those of the Acidobacterium division (Marilley and Aragno 1999). Low abundance and incidence of Gram-positive bacteria in soils at high altitudes were observed both in culture-dependent and culture-independent studies (Margesin et al. 2009). Gram-negative bacteria seem to be more competitive at the conditions prevailing at high altitudes due to their ability to be more tolerant to freeze–thaw cycles (Aislabie et al. 2006).

Among yeasts, *S. terricola*, *A. porosum* and *S. podzolica* represented the dominant species. These species were previously assigned to the genera *Trichosporon* (*T. porosum*) and *Cryptococcus* (*C. terricola* and *C. podzolicus*), which were reported to be dominant culturable genera in mountain and Alpine habitats (Turchetti et al. 2008, 2013, 2014; Buzzini et al. 2012; Buzzini and Margesin 2014). In particular, the species belonging to the former genus *Cryptococcus* have been observed in most soils (Botha 2006; Vishniac 2006). They were also found to inhabit densely vegetated soils, including forest, podzolic and sod-podzolic soils, in regions with tropical, temperate and cold climates (Vishniac 2006; Fonseca et al. 2011; Sugita 2011). Likewise, all yeast species found in this study, were also currently found as a part of yeast diversity in soil ecosystems (Golubev 1984; Maksimova and Cherov 2003; Wuczkowski and Prillinger 2004; Botha 2006; Golubtsova et al. 2006; Lynch and Thorn 2006; Vishniac 2006; Golubev and Scorzetti 2010; Mestre et al. 2011; Yurkov et al. 2011, 2012, 2016; Buzzini et al. 2012). As previously reported, soil yeasts can contribute to essential soil ecological processes, i.e. the mineralization of organic material and assimilation of carbon to produce biomass and energy. Some soil yeasts may also play a role in soil nitrogen and sulphur cycles and have the ability to solubilize insoluble phosphates making them more readily available for plants (Botha 2006, 2011).

Before its taxonomic update, *Cryptococcus* was a highly polyphyletic genus including species belonging to more than one order of *Tremellomycetes* (Fonseca et al. 2011). Accordingly, as the consequence of the update of *Tremellomycetes* taxonomy based on the results of phylogenetic analyses from a seven-genes dataset covering the majority of tremellomycetous yeasts (including closely related filamentous taxa), a number of species have been recently taxonomically reassigned to some newly described genera (Liu et al. 2016).

Four out of 36 species (about 11 %) could be regarded as potential novel yeast species according to their phylogenetic affiliation and sequence similarity with the closest related type strain. As for bacteria, these yet undescribed yeast taxa confirmed that forest sites can represent a reservoir of unexplored members of microbial communities. Their taxonomic assignment via polyphasic characterization is in progress.

PCA plots on the total bacterial diversity recovered at each site and each season (without distinction between psychrophiles and mesophiles) demonstrated site-specific biodiversity independent of the season (Fig. 3b): spring and autumn isolates from each site clustered together and apart from the remaining sites. However, community structures between strains isolated at 0 and 20 °C were different (Fig. 3a). Among the latter we observed that a few dominant culturable taxa were present at all sites in both seasons. This indicates the presence of a resilient culturable bacterial population that is not affected by season or altitude. In contrast, strains isolated at 0 °C from the submontane and the montane site were distinct from those from the subalpine and the alpine site in both seasons, which demonstrates differences in bacterial community composition at lower and higher altitudes. Similar results were found with non-culture based assessment of bacterial communities at the sites investigated in this study in spring: the highest bacterial richness and diversity was found at the site at the lowest altitude (Siles and Margesin 2016). Distinct microbial community structures correlating with altitude have been observed in subalpine and alpine environments (Lipson 2007), Himalayan slopes (Gangwar et al. 2009) and in Indian (Ma et al. 2004), Chinese (Xu et al. 2015) and Peruan mountains (Fierer et al. 2011) among others.

Diversity indexes reported clear differences in yeast community parameters for isolations at 4 and 20 °C at the four sites: at the lower temperature, the dominance of a few species was found, while at 20 °C a more uniform species distribution was observed (Table 3). Interestingly, PCA plots on the total yeast diversity recovered at each site and in each season (without distinction between psychrophiles and mesophiles) reported an apparent season-dependent biodiversity (Fig. 4b). The different distribution patterns observed between bacterial and yeasts community structure could be the consequence of the existence of different factors governing bacterial and yeast culturable communities in the studied soils. Our data indicate that the diversity of culturable yeasts was more susceptible to seasonal changes, in close agreement with the study of Turchetti et al. (2013). As reported by Botha (2006), growth and survival of a particular yeast in soil may not solely depend on the intrinsic abilities of the yeast, but is a cumulative result of a number of interactions within each soil microbial community. Combinations of abiotic factors have been reported to apparently explain only a part of the distribution of the predominant yeast species, whereas vegetation type could play the same role for orders (Vishniac 2006). In contrast, the diversity of culturable bacterial communities seemed to be more affected by temperature, which resulted in changes related to altitude and isolation temperature. A more recent study (Birkhofer et al. 2012) explored the relationship between soil properties and soil biota in managed grassland and forest soils and reported a general correlation of abundance and diversity patterns of fungi and soil fauna with soil properties. However, the extraction of variation, explained by location and land-use type, led to the conclusion that soil properties still explain significant proportions of variation in the abundance and diversity of soil biota.

Altogether, this study is the first report on seasonal and altitudinal changes considering both bacterial and yeast culturable diversity in Alpine forest soils. Distinct differences between culturable bacterial and yeast diversity related to altitude, season and isolation temperature were recognized. Our data also showed a remarkably high occurrence of potential novel bacterial species (more than 50 % of the retrieved 112 OTUs) and yeast species (about 11 % of the retrieved 36 species) that may be of ecological relevance at all altitudes. The percentage of potential novel yeast species observed in our study is comparable with that reported for unexplored diversity predicted for forest soils (Yurkov et al. 2011, 2012, 2016).

The application of higher coverage molecular techniques might give additional insights into the altitudinal and seasonal patterns of psychrophilic and mesophilic microbial communities at the investigated sites. For a better understanding of changing climate conditions (associated with altitude and seasonality) on soil microorganisms, more research is needed and especially important with regard to the consequences of the global change of climate on forest soils.

## Electronic supplementary material

Below is the link to the electronic supplementary material.

Supplementary material 1 (PDF 1097 kb)

## References

[CR1] Aislabie JM, Broady PA, Saul DJ (2006). Culturable aerobic heterotrophic bacteria from high altitude, high latitude soil of La Gorce Mountains (86 30′S, 147 W), Antarctica. Antarct Sci.

[CR2] Arenas FA, Pugin B, Henríquez NA, Arenas-Salinas MA, Diaz-Vasquez WA, Pozo MF, Munoz CM, Chasteen TG, Perez-Donoso JM, Vasquez CC (2014). Isolation, identification and characterization of highly tellurite-resistant, tellurite-reducing bacteria from Antarctica. Polar Sci.

[CR3] Birkhofer K, Schöning I, Alt F, Herold N, Klarner B, Maraun M, Marhan S, Oelmann Y, Wubet T, Yurkov A, Berner D, Buscot F, Daniel R, Diekötter T, Ehnes RB, Erdmann G, Fischer C, Foesel B, Groh J, Gutknecht J, Kandeler E, Lang C, Lohaus G, Meyer A, Nacke H, Näther A, Overmann J, Polle A, Pollierer MM, Scheu S, Schloter M, Schulze ED, Schulze W, Weinert J, Weisser WW, Wolters V, Schrumpf M (2012). General relationships between abiotic soil properties and soil biota across spatial scales and different land-use types. PLoS One.

[CR4] Bossio D, Scow K, Gunapala N, Graham K (1998). Determinants of soil microbial communities: effects of agricultural management, season, and soil type on phospholipid fatty acid profiles. Microb Ecol.

[CR5] Botha A, Rosa CA, Peter G (2006). Yeast in soil. Biodiversity and ecophysiology of yeasts.

[CR6] Botha A (2011). The importance and ecology of yeasts in soil. Soil Biol Biochem.

[CR8] Brockett BF, Prescott CE, Grayston SJ (2012). Soil moisture is the major factor influencing microbial community structure and enzyme activities across seven biogeoclimatic zones in western Canada. Soil Biol Biochem.

[CR9] Buzzini P, Margesin R (2014). Cold-adapted yeasts: biodiversity, adaptation strategies and biotechnological significance.

[CR10] Buzzini P, Branda E, Goretti M, Turchetti B (2012). Psychrophilic yeasts from worldwide glacial habitats: diversity, adaptation strategies and biotechnological potential. FEMS Microbiol Ecol.

[CR11] Caporaso JG, Kuczynski J, Stombaugh J, Bittinger K, Bushman FD, Costello EK, Fierer N, Peña AG, Goodrich JK, Gordon JI, Huttley GA, Kelley ST, Knights D, Koenig JE, Ley RE, Lozupone CA, McDonald D, Muegge BD, Pirrung M, Reeder J, Sevinsky JR, Turnbaugh PJ, Walters WA, Widmann J, Yatsunenko T, Zaneveld J, Knight R (2010). QIIME allows analysis of high-throughput community sequencing data. Nat Methods.

[CR12] Cavicchioli R (2006). Cold-adapted archaea. Nat Rev Microbiol.

[CR13] Delorme S, Lemanceau P, Christen R, Corberand T, Meyer J-M, Gardan L (2002). *Pseudomonas lini* sp. nov., a novel species from bulk and rhizospheric soils. Int J Syst Evol Microbiol.

[CR14] Diaz HF, Grosjean M, Graumlich L (2003). Climate variability and change in high elevation regions: past, present and future. Clim Change.

[CR15] Donachie SP, Foster JS, Brown MV (2007). Culture clash: challenging the dogma of microbial diversity. ISME J.

[CR16] Edgar RC (2010). Search and clustering orders of magnitude faster than BLAST. Bioinformatics.

[CR17] Feller G, Gerday C (2003). Psychrophilic enzymes: hot topics in cold adaptation. Nat Rev Microbiol.

[CR18] Fierer N, McCain CM, Meir P, Zimmermann M, Rapp JM, Silman MR, Knight R (2011). Microbes do not follow the elevational diversity patterns of plants and animals. Ecology.

[CR19] Fonseca A, Boekhout T, Fell JW, Kurtzman CP, Fell JW, Boekhout T (2011). *Cryptococcus* Vuillemin (1901). The yeasts: a taxonomic study.

[CR20] França L, Lopéz-Lopéz A, Rosselló-Móra R, da Costa MS (2015). Microbial diversity and dynamics of a groundwater and a still bottled natural mineral water. Environ Microbiol.

[CR21] Frey-Klett P, Burlinson P, Deveau A, Barret M, Tarkka M, Sarniguet A (2011). Bacterial-fungal interactions: hyphens between agricultural, clinical, environmental, and food microbiologists. Microbiol Mol Biol Rev.

[CR22] Gadanho M, Sampaio JP (2002). Polyphasic taxonomy of the basidiomycetous yeast genus *Rhodotorula*: *R. glutinis* sensu stricto and *R. dairensis* comb. nov. FEMS Yeast Res.

[CR23] Gangwar P, Alam SI, Bansod S, Singh L (2009). Bacterial diversity of soil samples from the western Himalayas, India. Can J Microbiol.

[CR24] Gavazov KS (2010). Dynamics of alpine plant litter decomposition in a changing climate. Plant Soil.

[CR25] Giri DD, Shukla PN, Kashyap S, Singh P, Kashyap AK, Pandey KD (2007). Variation in methanotrophic bacterial population along an altitude gradient at two slopes in tropical dry deciduous forest. Soil Biol Biochem.

[CR26] Golubev WI (1984). *Cryptococcus fuscescens* sp. nov. and a diagnostic key to the nitrate-positive species of the genus *Cryptococcus*. J Gen Appl Microbiol.

[CR27] Golubev WI, Scorzetti G (2010). *Rhodotorula rosulata* sp. nov., *Rhodotorula silvestris* sp. nov. and *Rhodotorula straminea* sp. nov., novel myo-inositol-assimilating yeast species in the *Microbotryomycetes*. Int J Syst Evol Microbiol.

[CR28] Golubtsova VY, Glushakova AM, Chernov IY (2006). The seasonal dynamics of yeast communities in the rhizosphere of soddy-podzolic soils. Euras Soil Sci.

[CR29] Hammer Ø, Ryan P, Harper D (2001). PAST: paleontological statistics software package for education and data analysis. Palaeontol Electron.

[CR30] Jing X, Wang YH, Chung HG, Mi ZR, Wang SP, Zeng H, He JS (2014). No temperature acclimation of soil extracellular enzymes to experimental warming in an alpine grassland ecosystem on the Tibetan Plateau. Biogeochemistry.

[CR31] Kuhn M, Margesin R, Schinner F, Marx J-C, Gerday C (2008). The climate of snow and ice as boundary condition for microbial life. Psychrophiles: from biodiversity to biotechnology.

[CR32] Li K, Bihan M, Yooseph S, Methé BA (2012). Analyses of the microbial diversity across the human microbiome. PLoS One.

[CR33] Libkind D, Brizzio S, Ruffini A, Gadanho M, van Broock M, Sampaio JP (2003). Molecular characterization of carotenogenic yeasts from aquatic environments in Patagonia, Argentina. Antonie Van Leeuwenhoek.

[CR34] Lipson DA (2007). Relationships between temperature responses and bacterial community structure along seasonal and altitudinal gradients. FEMS Microbiol Ecol.

[CR35] Lipson DA, Schmidt SK (2004). Seasonal changes in an alpine soil bacterial community in the Colorado Rocky Mountains. Appl Environ Microb.

[CR36] Liu X-Z, Wang Q-M, Göker M, Groenewald M, Kachalkin AV, Lumbsch HT, Millanes AM, Wedin M, Yurkov AM, Boekhout T, Bai FY (2016). Towards an integrated phylogenetic classification of the *Tremellomycetes*. Stud Mycol.

[CR37] Ludwig W, Strunk O, Westram R, Richter L, Meier H, Yadhukumar Buchner A, Lai T, Steppi S, Jobb G (2004). ARB: a software environment for sequence data. Nucl Acids Res.

[CR38] Lynch MDJ, Thorn RG (2006). Diversity of basidiomycetes in Michigan agricultural soils. Appl Env Microbiol.

[CR39] Ma XJ, Chen T, Zhang GS, Wang R (2004). Microbial community structure along an altitude gradient in three different localities. Folia Microbiol.

[CR40] Maksimova IA, Cherov IY (2003). Community structure of yeast fungi in forest biogeocenoses. Microbiology.

[CR41] Männistö MK, Häggblom MM (2006). Characterization of psychrotolerant heterotrophic bacteria from Finnish Lapland. Syst Appl Microbiol.

[CR42] Männistö MK, Tiirola M, Häggblom MM (2007). Bacterial communities in Arctic fjelds of Finnish Lapland are stable but highly pH-dependent. FEMS Microbiol Ecol.

[CR43] Margesin R (2009). Effect of temperature on growth parameters of psychrophilic bacteria and yeasts. Extremophiles.

[CR44] Margesin R, Lütz C (2012). Psychrophilic microorganisms in alpine soils. Plants in alpine regions.

[CR45] Margesin R, Miteva V (2011). Diversity and ecology of psychrophilic microorganisms. Res Microbiol.

[CR46] Margesin R, Jud M, Tscherko D, Schinner F (2009). Microbial communities and activities in alpine and subalpine soils. FEMS Microbiol Ecol.

[CR47] Margesin R, Minerbi S, Schinner F (2014). Long-term monitoring of soil microbiological activities in two forest sites in South Tyrol in the Italian Alps. Microbes Environ.

[CR48] Marilley L, Aragno M (1999). Phylogenetic diversity of bacterial communities differing in degree of proximity of *Lolium perenne* and *Trifolium repens* roots. Appl Soil Ecol.

[CR49] Marmur J (1963). A procedure for the isolation of deoxyribonucleic acid from microorganisms. Meth Enzym.

[CR50] Mestre MC, Rosa CA, Safar SVB, Libkind D, Fontenla SB (2011). Yeast communities associated with the bulk-soil, rhizosphere and ectomycorrhizosphere of a *Nothofagus pumilio* forest in northwestern Patagonia, Argentina. FEMS Microbiol Ecol.

[CR51] Meyer A, Lipson D, Martin A, Schadt C, Schmidt S (2004). Molecular and metabolic characterization of cold-tolerant alpine soil *Pseudomonas* sensu stricto. Appl Environ Microbiol.

[CR52] Morita RY (1975). Psychrophilic bacteria. Bacteriol Rev.

[CR53] Nagahama T, Hamamoto M, Nakase T, Takami H, Horikoshi K (2001). Distribution and identification of red yeasts in deep-sea environments around the northwest Pacific Ocean. Antonie Van Leeuwenhoek.

[CR54] Nagahama T, Hamamoto M, Nakase T, Takaki Y, Horikoshi K (2003). *Cryptococcus surugaensis* sp. nov., a novel yeast species from sediment collected on the deep-sea floor of Suruga Bay. Int J Syst Evol Microbiol.

[CR55] Nemergut DR, Costello EK, Meyer AF, Pescador MY, Weintraub MN, Schmidt SK (2005). Structure and function of alpine and arctic soil microbial communities. Res Microbiol.

[CR56] Pedrós-Alió C (2006). Marine microbial diversity: can it be determined?. Trends Microbiol.

[CR57] Pruesse E, Peplies J, Glöckner FO (2012). SINA: accurate high-throughput multiple sequence alignment of ribosomal RNA genes. Bioinformatics.

[CR58] Rainey FA, Ward-Rainey N, Kroppenstedt RM, Stackebrandt E (1996). The genus *Nocardiopsis* represents a phylogenetically coherent taxon and a distinct actinomycete lineage: proposal of *Nocardiopsaceae* fam. nov. Int J Syst Bacteriol.

[CR59] Rappé MS, Giovannoni SJ (2003). The uncultured microbial majority. Annu Rev Microbiol.

[CR60] Rasche F, Knapp D, Kaiser C, Koranda M, Kitzler B, Zechmeister-Boltenstern S, Richter A, Sessitsch A (2011). Seasonality and resource availability control bacterial and archaeal communities in soils of a temperate beech forest. ISME J.

[CR61] Rosselló-Móra R (2012). Towards a taxonomy of Bacteria and Archaea based on interactive and cumulative data repositories. Environ Microbiol.

[CR62] Rousk J, Brookes PC, Bååth E (2010). The microbial PLFA composition as affected by pH in an arable soil. Soil Biol Biochem.

[CR63] Sampaio JP, Gadanho M, Santos S, Duarte F, Pais C, Fonseca A, Fell JW (2001). Polyphasic taxonomy of the basidiomycetous yeast genus *Rhodosporidium*: *Rhodosporidium kratochvilovae* and related anamorphic species. Int J Syst Evol Microbiol.

[CR64] Shen C, Xiong JB, Zhang HY, Feng YZ, Lin XG, Li XY, Liang WJ, Chu HY (2013). Soil pH drives the spatial distribution of bacterial communities along elevation on Changbai Mountain. Soil Biol Biochem.

[CR65] Siles JA, Margesin R (2016). Abundance and diversity of bacterial, archaeal and fungal communities along an altitudinal gradient in Alpine forest soils: what are the driving factors?. Microb Ecol.

[CR66] Siles JA, Cajthaml T, Minerbi S, Margesin R (2016). Effect of altitude and season on microbial activity, abundance and community structure in Alpine forest soils. FEMS Microbiol Ecol.

[CR67] Smit E, Leeflang P, Gommans S, van den Broek J, van Mil S, Wernars K (2001). Diversity and seasonal fluctuations of the dominant members of the bacterial soil community in a wheat field as determined by cultivation and molecular methods. Appl Environ Microbiol.

[CR68] Stackebrandt E, Ebers J (2006). Taxonomic parameters revisited: tarnished gold standards. Microbiol Today.

[CR69] Stackebrandt E, Goebel B (1994). Taxonomic note: a place for DNA-DNA reassociation and 16S rRNA sequence analysis in the present species definition in bacteriology. Int J Syst Bacteriol.

[CR70] Stamatakis A (2006). RAxML-VI-HPC: maximum likelihood-based phylogenetic analyses with thousands of taxa and mixed models. Bioinformatics.

[CR71] Sugita T, Kurtzman CP, Fell JW, Boekhout T (2011). *Trichosporon* Behrend (1890). The yeasts: a taxonomic study.

[CR72] Tamura K, Dudley J, Nei M, Kumar S (2007). MEGA4: molecular evolutionary genetics analysis (MEGA) software version 4.0. Mol Biol Evol.

[CR73] Turchetti B, Buzzini P, Goretti M, Branda E, Diolaiuti G, D’Agata C, Smiraglia C, Vaughan-Martini A (2008). Psychrophilic yeasts in glacial environments of Alpine glaciers. FEMS Microbiol Ecol.

[CR74] Turchetti B, Goretti M, Branda E, Diolaiuti G, D’Agata C, Smiraglia C, Onofri A, Buzzini P (2013). Influence of abiotic variables on culturable yeast diversity in two distinct Alpine glaciers. FEMS Microbiol Ecol.

[CR75] Turchetti B, Goretti M, Buzzini P, Margesin R, Buzzini P, Margesin R (2014). Cold-adapted yeasts in Alpine and Apennine glaciers. Cold-adapted yeasts: biodiversity, adaptation strategies and biotechnological significance.

[CR76] Uchida M, Nakatsubo T, Kasai Y, Nakane K, Horikoshi T (2000). Altitudinal differences in organic matter mass loss and fungal biomass in a subalpine coniferous forest, Mt. Fuji, Japan. Arct Antarct Alp Res.

[CR77] Urbanová M, Šnajdr J, Baldrian P (2015). Composition of fungal and bacterial communities in forest litter and soil is largely determined by dominant trees. Soil Biol Biochem.

[CR78] Vishniac HS (2006). A multivariate analysis of soil yeasts isolated from a latitudinal gradient. Microb Ecol.

[CR79] Wu Y, Ma B, Zhou L, Wang H, Xu J, Kemmitt S, Brookes PC (2009). Changes in the soil microbial community structure with latitude in eastern China, based on phospholipid fatty acid analysis. Appl Soil Ecol.

[CR80] Wuczkowski M, Prillinger H (2004). Molecular identification of yeasts from soils of the alluvial forest national park along the river Danube downstream of Vienna, Austria (“Nationalpark Donauauen”). Microbiol Res.

[CR81] Xu ZW, Yu GR, Zhang XY, Ge JP, He NP, Wang QF, Wang D (2015). The variations in soil microbial communities, enzyme activities and their relationships with soil organic matter decomposition along the northern slope of Changbai Mountain. Appl Soil Ecol.

[CR82] Yarza P, Richter M, Peplies J, Euzeby J, Amann R, Schleifer KH, Ludwig W, Glokner FO, Rosselló-Móra R (2008). The all-species living tree project: a 16S rRNA-based phylogenetic tree of all sequenced type strains. Syst Appl Microbiol.

[CR83] Yarza P, Yilmaz P, Pruesse E, Glöckner FO, Ludwig W, Schleifer KH, Whitman WB, Euzeby J, Amann R, Rosselló-Móra R (2014). Uniting the classification of cultured and uncultured bacteria and archaea using 16S rRNA gene sequences. Nat Rev Microbiol.

[CR84] Yergeau E, Bokhorst S, Huiskes AH, Boschker HT, Aerts R, Kowalchuk GA (2007). Size and structure of bacterial, fungal and nematode communities along an Antarctic environmental gradient. FEMS Microbiol Ecol.

[CR85] Yung CC, Chan Y, Lacap DC, Pérez-Ortega S, de Los Rios-Murillo A, Lee CK, Cary SC, Pointing SB (2014). Characterization of chasmoendolithic community in Miers Valley, McMurdo Dry Valleys, Antarctica. Microb Ecol.

[CR86] Yurkov AM, Kemler M, Begerow D (2011). Species accumulation curves and incidence-based species richness estimators to appraise the diversity of cultivable yeasts from beech forest soils. PLoS One.

[CR87] Yurkov AM, Kemler M, Begerow D (2012). Assessment of yeast diversity in soils under different management regimes. Fungal Ecol.

[CR88] Yurkov AM, Röhl O, Pontes A, Carvalho C, Maldonado C, Sampaio JP (2016). Local climatic conditions constrain soil yeast diversity patterns in Mediterranean forests, woodlands and scrub biome. FEMS Yeast Res.

[CR89] Zumsteg A, Schmutz S, Frey B (2013). Identification of biomass utilizing bacteria in a carbon-depleted glacier forefield soil by the use of 13C DNA stable isotope probing. Environ Microbiol Rep.

