# Supplementary material for: Seasonal and altitudinal changes of culturable bacterial and yeast diversity in Alpine forest soils
Source: Extremophiles. 2016 Sep 12;20(6):855–73. doi: 10.1007/s00792-016-0874-2 (PMC5085987; doi:10.1007/s00792-016-0874-2)
Supplement: Supplementary file 1 — Supplementary material 1 (PDF 1097 kb) [file 792_2016_874_MOESM1_ESM.pdf]

Electronic supplementary material

Seasonal and altitudinal changes of culturable bacterial and yeast diversity in Alpine forest soils

Luís França[1], Ciro Sannino[2], Benedetta Turchetti[2], Pietro Buzzini[2], Rosa Margesin[1*]

[1] Institute of Microbiology, University of Innsbruck, Technikerstrasse 25, 6020 Innsbruck, Austria

[2] Department of Agricultural, Food and Environmental Sciences, Industrial Yeasts Collection DBVPG, University of Perugia, Borgo XX Giugno 74, 06121 Perugia, Italy

*Correspondence to: Rosa.Margesin@uibk.ac.at

Journal: Extremophiles

Fig. S1 Rarefaction curves for the observed number of bacterial OTUs (top) and yeast species (bottom) in the dataset of isolates.

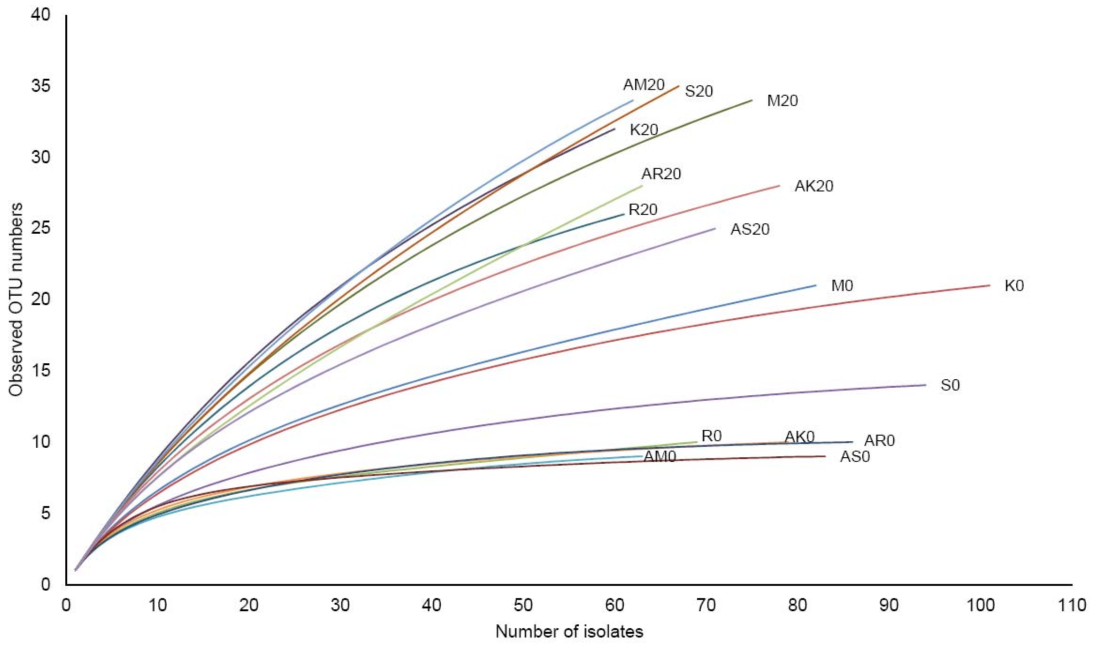

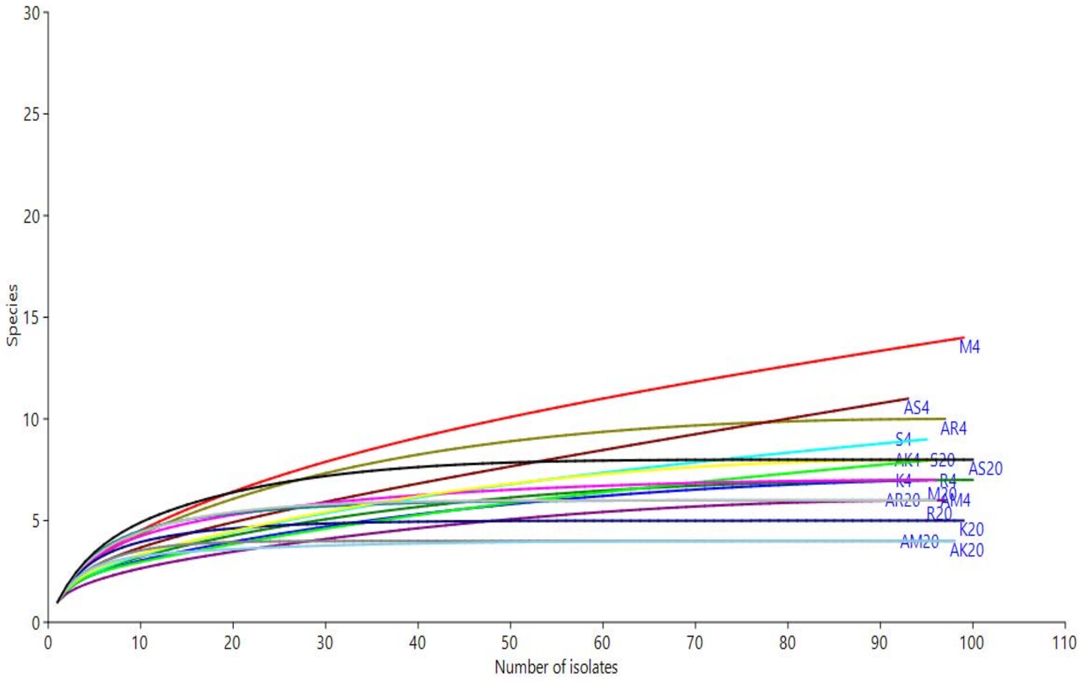

Fig. S2 Maximum likelihood (ML) tree showing the phylogenetic relationships of the 16S rRNA gene sequences of the representatives of each bacterial OTU with the most closely related reference sequences of the LTP database 119. The ML tree was constructed using sequences longer than 1,284 bp with a 40% conservational filter. The scale bar infers 10 nucleotide substitutions per 100 sequenced nucleotides.

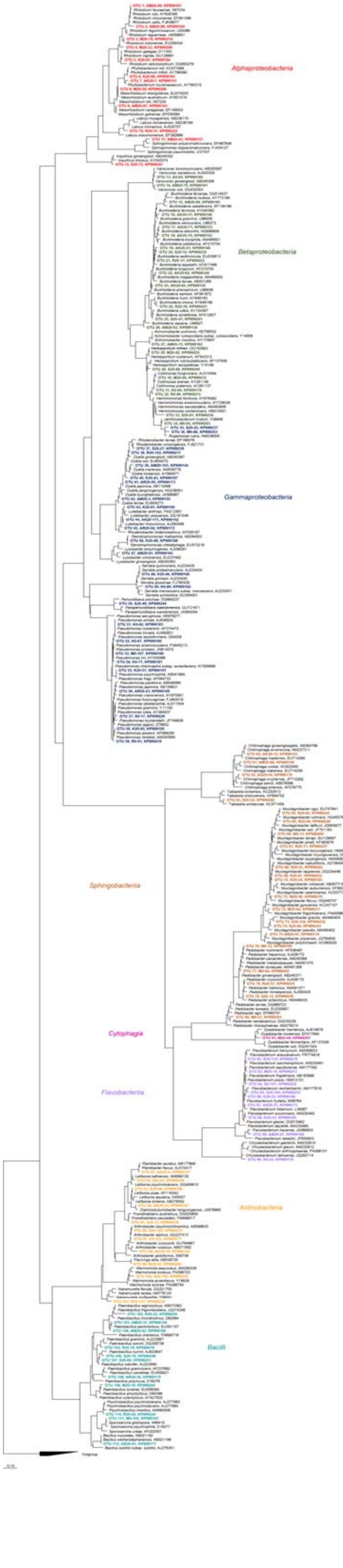
Alphaproteobacteria
Betaproteobacteria
Gammaproteobacteria
Sphingobacteriia
Cytophagia
Flavobacteriia
Actinobacteria
Bacilli
Outgroup

Fig. S3 Relative proportions of the different bacterial classes recovered at 0°C and 20ºC in spring and autumn seasons.

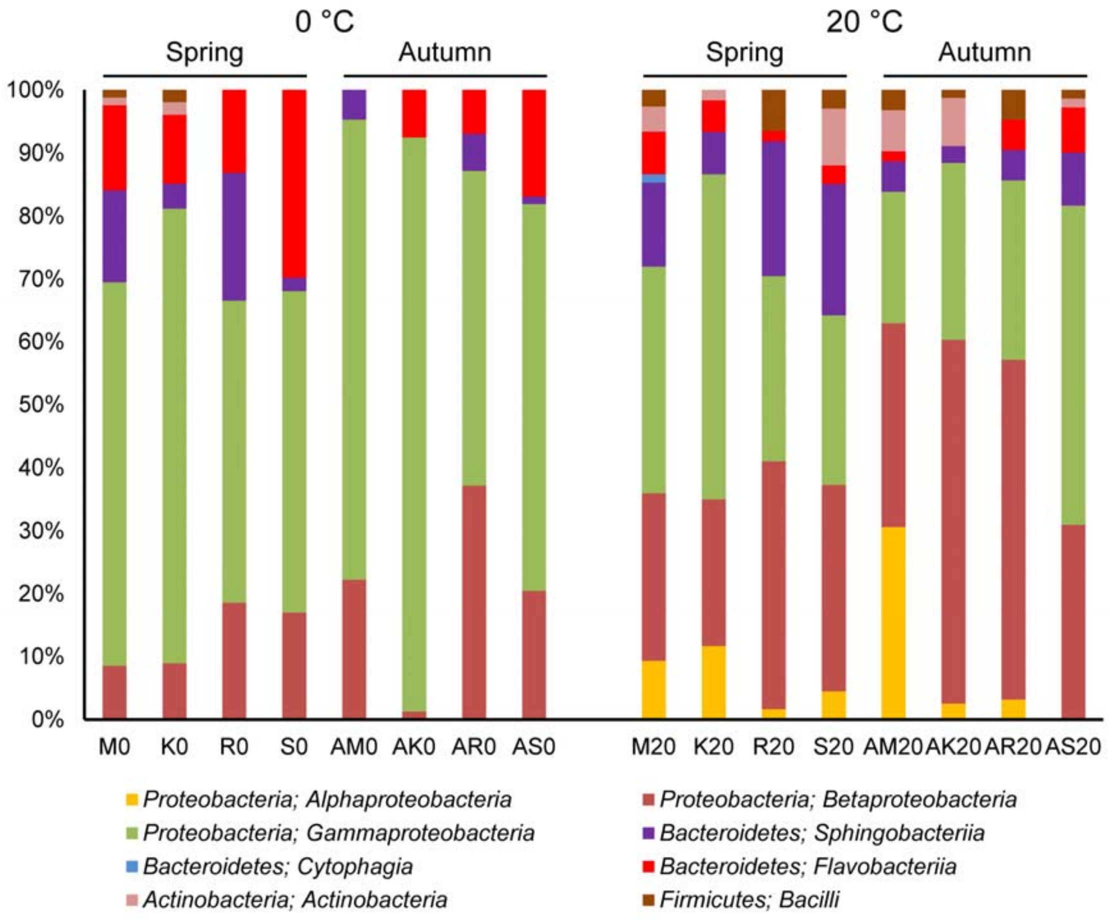

Fig. S4 Neighbor-joining tree of the classes Saccharomycetes and Dothideomycetes, phylum Ascomycota showing the phylogenetic relationship of the Internal Transcribed Spacers (ITS1 and 2 regions) including the 5.8S rRNA gene sequences of the representative yeast strains isolated and the type strains sequences obtained from GenBank database. The tree was rooted with *Saccharomyces cerevisiae* and *Zygosaccharomyces rouxii* type strains. Bootstrap percentages from 1000 replications are shown on the branches (values below 50 % are not shown). GenBank accession numbers of the sequences are indicated after strain numbers.

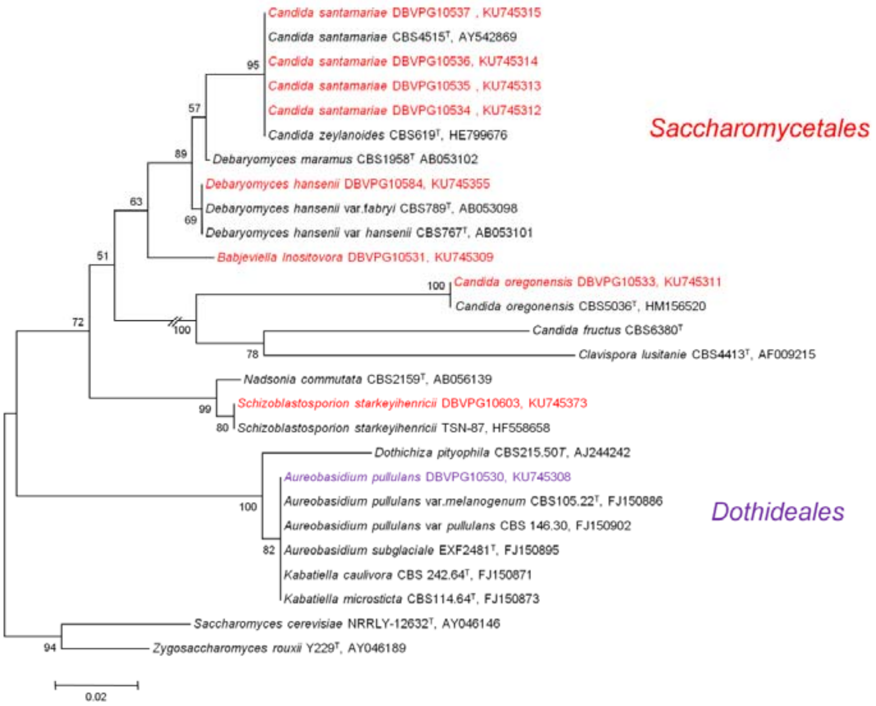

Fig. S5 Neighbor-joining tree of the class Tremellomycetes: order *Tremellales* (Fig. S5A), order *Trichosporonales* (Fig. S5B), order *Holtermanniales* (Fig. S5C), order *Filobasidiales* (Fig. S5D), order *Cystofilobasidiales* (Fig. S5E), and class *Microbotryomycetes* (Fig. S5F), phylum Basidiomycota, showing the phylogenetic relationship of the Internal Transcribed Spacers (ITS1 and 2 regions) including the 5.8S rRNA gene sequences of the representative yeast strains isolated and the type strains sequences obtained from GenBank database. Bootstrap percentages from 1000 replications are shown on the branches (values below 50% are not shown). GenBank accession numbers of the sequences are indicated after strain numbers.

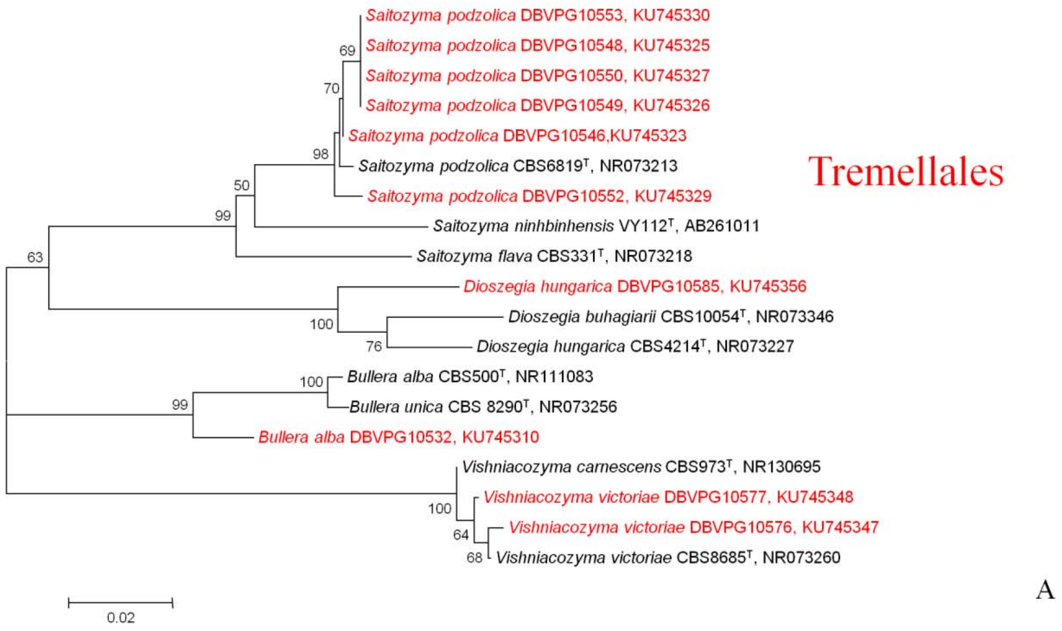

Saitozyma podzolica DBVPG10553, KU745330
Saitozyma podzolica DBVPG10548, KU745325
Saitozyma podzolica DBVPG10550, KU745327
Saitozyma podzolica DBVPG10549, KU745326
Saitozyma podzolica DBVPG10546,KU745323
Saitozyma podzolica CBS6819T, NR073213
Saitozyma podzolica DBVPG10552, KU745329
Saitozyma ninhbinhensis VY112T, AB261011
Saitozyma flava CBS331T, NR073218
Dioszegia hungarica DBVPG10585, KU745356
Dioszegia buhagiarii CBS10054T, NR073346
Dioszegia hungarica CBS4214T, NR073227
Bullera alba CBS500T, NR111083
Bullera unica CBS 8290T, NR073256
Bullera alba DBVPG10532, KU745310
Vishniacozyma carnescens CBS973T, NR130695
Vishniacozyma victoriae DBVPG10577, KU745348
Vishniacozyma victoriae DBVPG10576, KU745347
Vishniacozyma victoriae CBS8685T, NR073260
69
70
98
50
99
63
100
76
100
99
100
64
68
0.02
Tremellales
A

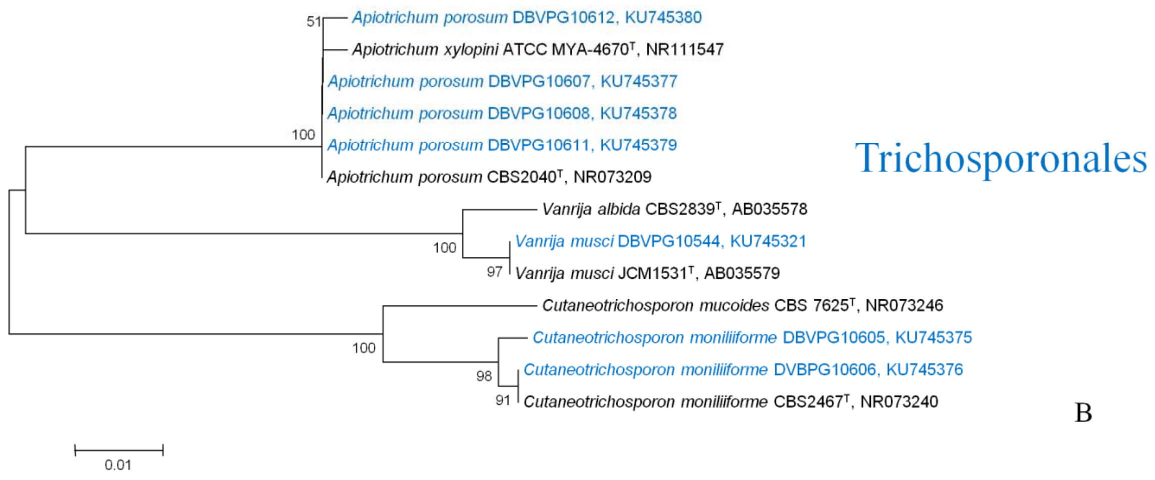

Apiotrichum porosum DBVPG10612, KU745380
Apiotrichum xylopini ATCC MYA-4670T, NR111547
Apiotrichum porosum DBVPG10607, KU745377
Apiotrichum porosum DBVPG10608, KU745378
Apiotrichum porosum DBVPG10611, KU745379
Apiotrichum porosum CBS2040T, NR073209
Vanrija albida CBS2839T, AB035578
Vanrija musci DBVPG10544, KU745321
Vanrija musci JCM1531T, AB035579
Cutaneotrichosporon mucoides CBS 7625T, NR073246
Cutaneotrichosporon moniliiforme DBVPG10605, KU745375
Cutaneotrichosporon moniliiforme DVBPG10606, KU745376
Cutaneotrichosporon moniliiforme CBS2467T, NR073240
51
100
100
97
100
98
91
0.01
Trichosporonales
B

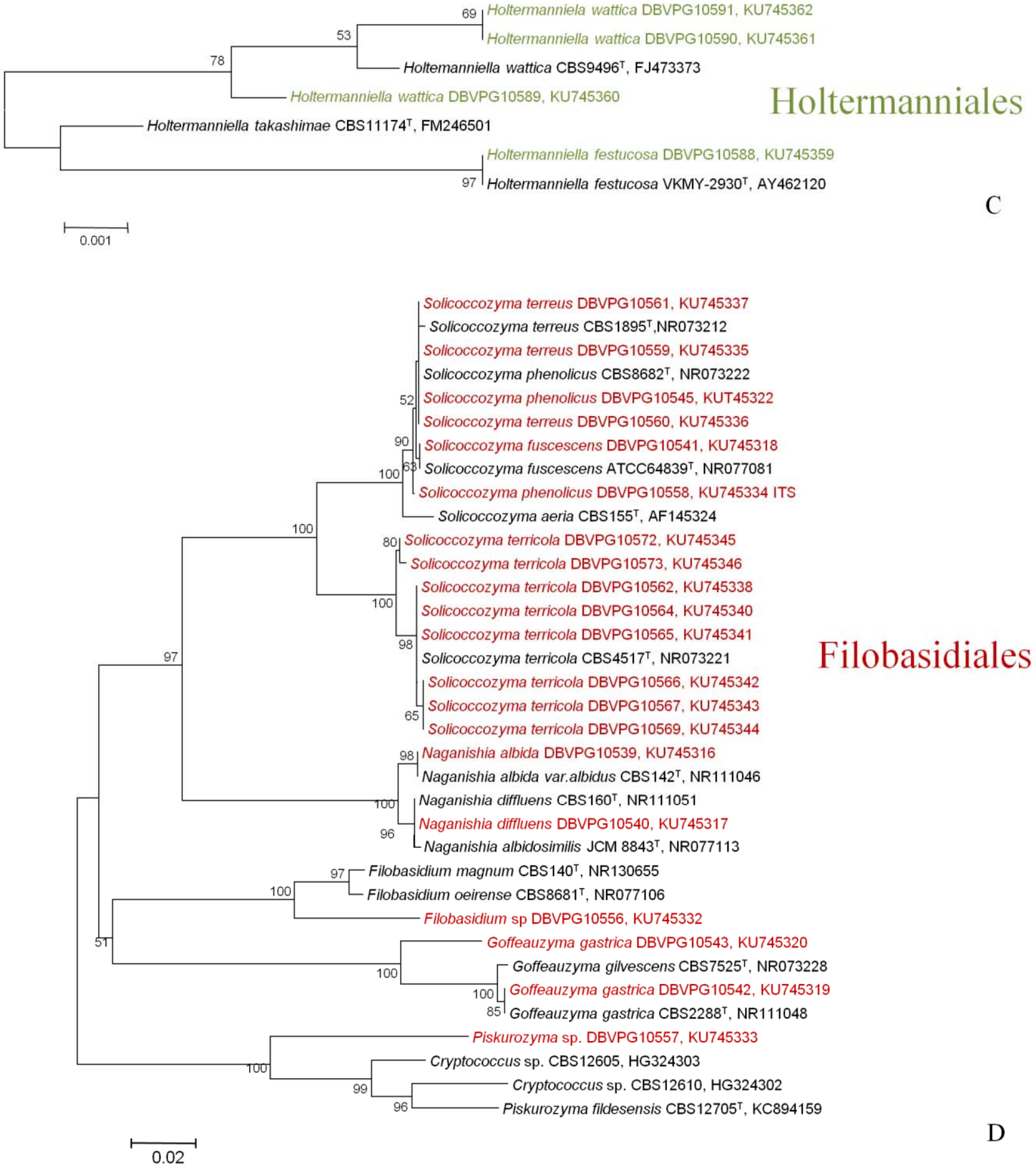
Holtermanniela wattica DBVPG10591, KU745362
Holtermanniela wattica DBVPG10590, KU745361
Holtemanniella wattica CBS9496T, FJ473373
Holtermanniela wattica DBVPG10589, KU745360
Holtermanniella takashimae CBS11174T, FM246501
Holtermanniella festucosa DBVPG10588, KU745359
Holtermanniella festucosa VKMY-2930T, AY462120
69
53
78
97
0.001
Holtermanniales
C
Solicoccozyma terreus DBVPG10561, KU745337
Solicoccozyma terreus CBS1895T,NR073212
Solicoccozyma terreus DBVPG10559, KU745335
Solicoccozyma phenolicus CBS8682T, NR073222
Solicoccozyma phenolicus DBVPG10545, KUT45322
Solicoccozyma terreus DBVPG10560, KU745336
Solicoccozyma fuscescens DBVPG10541, KU745318
Solicoccozyma fuscescens ATCC64839T, NR077081
Solicoccozyma phenolicus DBVPG10558, KU745334 ITS
Solicoccozyma aeria CBS155T, AF145324
Solicoccozyma terricola DBVPG10572, KU745345
Solicoccozyma terricola DBVPG10573, KU745346
Solicoccozyma terricola DBVPG10562, KU745338
Solicoccozyma terricola DBVPG10564, KU745340
Solicoccozyma terricola DBVPG10565, KU745341
Solicoccozyma terricola CBS4517T, NR073221
Solicoccozyma terricola DBVPG10566, KU745342
Solicoccozyma terricola DBVPG10567, KU745343
Solicoccozyma terricola DBVPG10569, KU745344
Naganishia albida DBVPG10539, KU745316
Naganishia albida var.albidus CBS142T, NR111046
Naganishia diffluens CBS160T, NR111051
Naganishia diffluens DBVPG10540, KU745317
Naganishia albidosimilis JCM 8843T, NR077113
Filobasidium magnum CBS140T, NR130655
Filobasidium oeirense CBS8681T, NR077106
Filobasidium sp DBVPG10556, KU745332
Goffeauzyma gastrica DBVPG10543, KU745320
Goffeauzyma gilvescens CBS7525T, NR073228
Goffeauzyma gastrica DBVPG10542, KU745319
Goffeauzyma gastrica CBS2288T, NR111048
Piskurozyma sp. DBVPG10557, KU745333
Cryptococcus sp. CBS12605, HG324303
Cryptococcus sp. CBS12610, HG324302
Piskurozyma fildesensis CBS12705T, KC894159
52
90
63
100
100
80
100
98
65
97
98
100
96
97
100
51
100
100
85
100
99
96
0.02
Filobasidiales
D

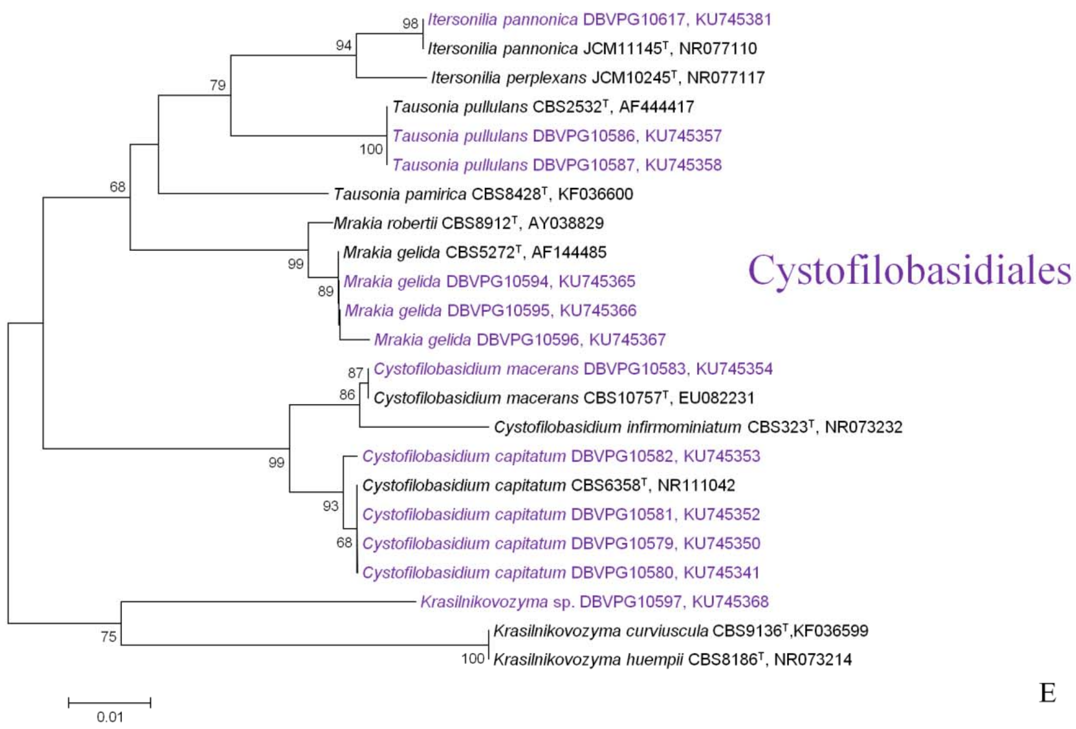
Itersonilia pannonica DBVPG10617, KU745381
Itersonilia pannonica JCM11145T, NR077110
Itersonilia perplexans JCM10245T, NR077117
Tausonia pullulans CBS2532T, AF444417
Tausonia pullulans DBVPG10586, KU745357
Tausonia pullulans DBVPG10587, KU745358
Tausonia pamirica CBS8428T, KF036600
Mrakia robertii CBS8912T, AY038829
Mrakia gelida CBS5272T, AF144485
Mrakia gelida DBVPG10594, KU745365
Mrakia gelida DBVPG10595, KU745366
Mrakia gelida DBVPG10596, KU745367
Cystofilobasidium macerans DBVPG10583, KU745354
Cystofilobasidium macerans CBS10757T, EU082231
Cystofilobasidium infirmominiatum CBS323T, NR073232
Cystofilobasidium capitatum DBVPG10582, KU745353
Cystofilobasidium capitatum CBS6358T, NR111042
Cystofilobasidium capitatum DBVPG10581, KU745352
Cystofilobasidium capitatum DBVPG10579, KU745350
Cystofilobasidium capitatum DBVPG10580, KU745341
Krasilnikovozyma sp. DBVPG10597, KU745368
Krasilnikovozyma curviuscula CBS9136T,KF036599
Krasilnikovozyma huempii CBS8186T, NR073214
98
94
79
100
68
99
89
87
86
99
93
68
75
100
0.01
Cystofilobasidiales

E

Fig. S6 Relative proportions of the different yeast species isolated at 4°C and 20°°C in spring and autumn seasons. Only yeast genera with more than three strains are shown. Dominant taxa present in all data subsets (at each site, in each season and at each isolation temperature) are boxed.

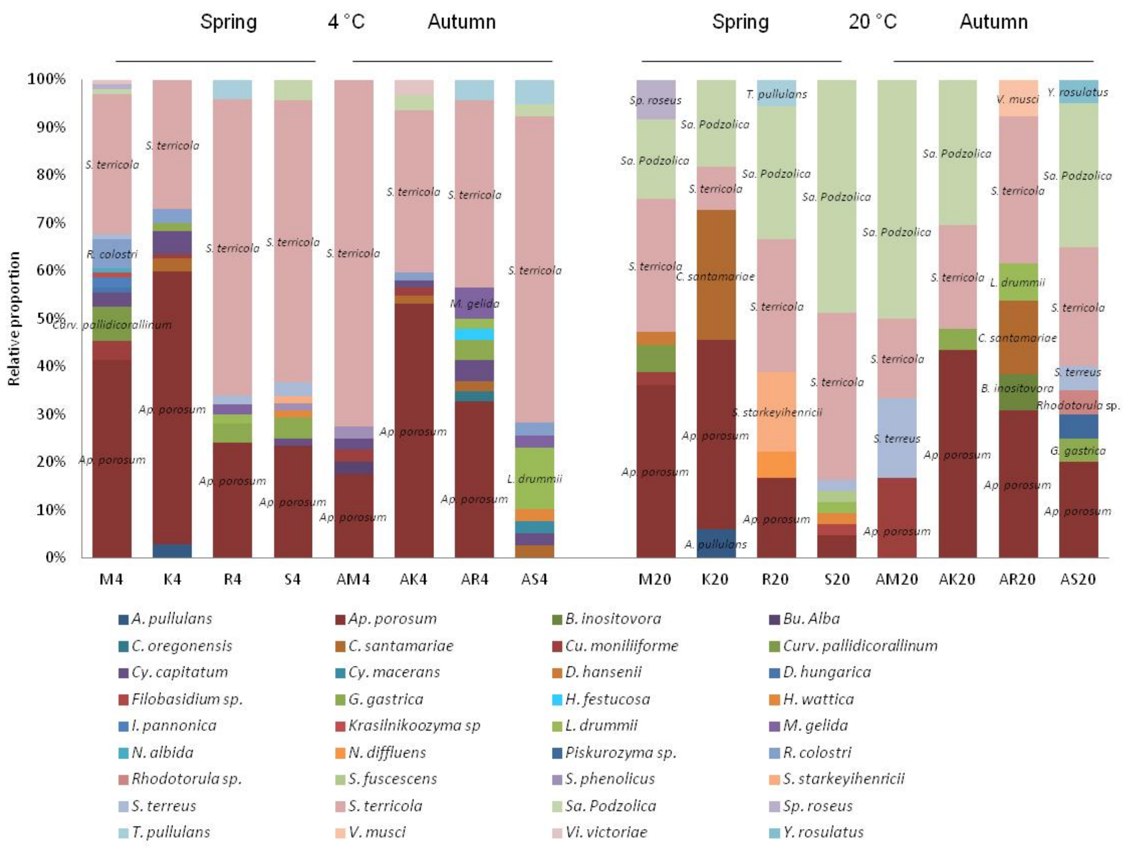

Table S1 Relative abundance (%) of the bacterial OTUs isolated at 0°C and 20 ºC in spring and autumn. Both the phylogenetic affiliation and the similarity value to the closest reference sequence in LTP 119 database are shown. The abundance is presented in gradient color according to percentages of the total number of isolates in a subset of datae. Closest relative identity values below 98% are marked in red.

			0 ºC								20 ºC									
			Spring				Autumn				Spring				Autumn					
Closest Relative, accession number	Closest relative identity (%)	OTU ID	M0 (n=82)	K0 (n=101)	R0 (n=69)	S0 (n=94)	AM0 (n=63)	AK0 (n=79)	AR0 (n=86)	AS0 (n=83)	M20 (n=75)	K20 (n=60)	R20 (n=61)	S20 (n=67)	AM20 (n=62)	AK20 (n=78)	AR20 (n=63)	AS20 (n=71)	Representative isolate, accession number	phylogenetic afiliation
Rhizobium leucaenae , X67234	99.8	1	-	-	-	-	-	-	-	-	2.7	3.3	-	-	12.9	1.3	1.6	-	AM20-94, KP899167	*Alphaproteobacteria*
Rhizobium vallis , FJ839677	97.8	2	-	-	-	-	-	-	-	-	-	-	-	-	1.6	-	-	-	AM20-88, KP899164	
Rhizobium tubonense , EU256434	99.8	3	-	-	-	-	-	-	-	-	1.3	-	-	-	3.2	-	-	-	M20-76, KP899214	
Rhizobium tubonense , EU256434	97.4	4	-	-	-	-	-	-	-	-	1.3	-	-	-	-	-	-	-	M20-53, KP899209	
Rhizobium vignae , GU128881	99.7	5	-	-	-	-	-	-	-	-	-	1.7	-	-	-	-	-	-	K20-87, KP899194	
Phyllobacterium trifolii , AY786080	99.8	6	-	-	-	-	-	-	-	-	-	1.7	-	-	1.6	-	-	-	K20-86, KP899193	
Phyllobacterium myrsinacearum , AY785315	99.0	7	-	-	-	-	-	-	-	-	1.3	-	-	-	-	1.3	-	-	AK20-7, KP899151	
Mesorhizobium shangrilense , EU074203	100.0	8	-	-	-	-	-	-	-	-	2.7	5.0	-	3.0	6.5	-	1.6	-	M20-50, KP899208	
Mesorhizobium caraganae , EF149003	99.8	9	-	-	-	-	-	-	-	-	-	-	-	-	1.6	-	-	-	AM20-87, KP899163	
Labrys monachus , AJ535707	97.3	10	-	-	-	-	-	-	-	-	-	-	1.6	-	-	-	-	-	R20-31, KP899222	
Sphingomonas polyaromaticivorans , EF467848	97.7	11	-	-	-	-	-	-	-	-	-	-	-	-	3.2	-	-	-	AM20-41, KP899157	
Inquilinus gingengisoli , AB245352	98.6	12	-	-	-	-	-	-	-	-	-	-	-	1.5	-	-	-	-	S20-72, KP899247	
Variovorax boronicumulans , AB300597	99.5	13	-	2.0	-	-	-	-	-	-	-	-	-	-	3.2	-	1.6	4.2	K0-93, KP899184	*Betaproteobacteria*
Variovorax soli , DQ432053	98.9	14	-	-	-	-	-	-	-	-	-	1.7	-	-	1.6	-	-	-	AM20-70, KP899161	
Burkholderia ferrariae , DQ514537	97.6	15	-	-	-	-	-	-	-	-	-	-	-	-	3.2	-	-	-	AM20-69, KP899160	
Burkholderia graminis , U96939	97.7	16	-	-	-	-	-	-	-	-	-	-	-	-	3.2	3.9	-	-	AK20-31, KP899146	
Burkholderia dilworthii , HQ698908	98.7	17	-	-	-	-	-	-	-	-	-	1.7	3.3	4.5	1.6	11.5	1.6	4.2	AK20-71, KP899153	
Burkholderia dilworthii , HQ698908	98.1	18	-	-	-	-	-	-	-	-	-	-	-	-	-	1.3	4.8	-	AK20-70, KP899152	
Burkholderia sediminicola , EU035613	98.2	19	-	-	-	-	-	-	-	-	-	1.7	-	-	1.6	2.6	-	-	AK20-51, KP899148	
Burkholderia sediminicola , EU035613	99.0	20	1.2	-	-	-	-	-	-	-	-	-	-	1.5	-	-	3.2	-	S20-12, KP899235	
Burkholderia sediminicola , EU035613	99.6	21	-	-	-	-	-	-	-	-	4.0	-	4.9	-	1.6	9.0	-	-	R20-37, KP899223	
Burkholderia megapolitana , AM489502	98.2	22	-	-	-	-	-	-	-	-	-	3.3	-	-	-	2.6	1.6	-	AK20-62, KP899149	
Burkholderia phenazinium , U96936	99.2	23	-	-	-	-	-	-	-	-	-	-	1.6	-	-	3.9	-	-	AK20-65, KP899150	
Burkholderia choica , AY949196	98.7	24	-	-	-	-	-	-	-	-	-	1.7	1.6	-	-	-	1.6	-	R20-30, KP899221	
Burkholderia sordidicola , AF512827	98.5	25	6.1	1.0	-	-	1.6	-	-	-	2.7	5.0	4.9	4.5	11.3	16.7	15.9	1.4	S20-41, KP899241	
Burkholderia cepacia , U96927	97.2	26	-	-	-	-	-	-	-	-	-	-	-	1.5	1.6	-	1.6	-	AM20-63, KP899158	
Achromobacter xylosoxidans , Y14908	98.8	27	-	-	-	-	-	-	-	-	-	-	-	-	3.2	-	-	-	AM20-72, KP899162	
Herbaspirillum hiltneri , DQ150563	99.3	28	-	1.0	-	-	-	-	-	-	1.3	-	-	-	-	-	-	-	M20-42, KP899205	
Herbaspirillum seropedicae , Y10146	97.9	29	-	-	-	-	-	-	-	-	-	-	-	1.5	-	-	-	-	S20-88, KP899249	
Collimonas fungivorans , AJ310394	99.8	30	-	-	-	1.1	-	-	-	-	5.3	1.7	6.6	1.5	-	2.6	-	1.4	M20-66, KP899212	
Collimonas pratensis , AY281137	99.9	31	-	3.0	15.9	13.8	20.6	1.3	37.2	18.1	13.3	5.0	16.4	16.4	-	3.9	20.6	18.3	K0-60, KP899179	
Collimonas pratensis , AY281137	97.5	32	-	-	1.5	-	-	-	-	-	-	-	-	-	-	-	-	-	R0-86, KP899215	
Herminiimonas saxobsidens , AM493906	96.6	33	-	-	-	-	-	-	-	-	-	-	-	1.5	-	-	-	-	S20-91, KP899250	
Janthinobacterium lividum , Y08846	98.9	34	1.2	2.0	1.5	2.1	-	-	-	2.4	-	1.7	-	-	-	-	1.6	1.4	M0-95, KP899203	
Rugamonas rubra , HM038005	98.3	35	-	-	-	-	-	-	-	-	-	-	-	1.5	-	-	-	1.4	S20-25, KP899237	*Gammaproteobacteria*
Rugamonas rubra , HM038005	99.0	36	1.2	-	-	-	-	1.3	-	-	-	-	-	-	-	-	-	-	M0-96, KP899253	
Rhodanobacter umsongensis , FJ821731	99.1	37	-	-	-	-	-	-	-	-	-	-	-	1.5	-	-	-	1.4	S20-27, KP899238	
Dyella ginsengisoli , AB245367	98.6	38	-	-	-	-	-	-	-	-	-	-	6.6	-	-	-	4.8	-	R20-102, KP899217	
Dyella marensis , AM939778	98.3	39	-	-	-	-	-	-	-	-	4.0	1.7	-	-	3.2	-	-	-	AM20-103, KP899154	
Dyella japonica , AB110498	98.3	40	-	-	-	-	-	-	-	-	-	1.7	-	-	3.2	-	-	-	K20-43, KP899187	
Dyella japonica , AB110498	99.9	41	-	-	-	-	-	-	-	-	1.3	-	-	-	1.6	-	1.6	-	AR20-95, KP899173	
Dyella terrae , EU604273	99.1	42	-	-	-	-	-	-	-	-	-	-	-	-	1.6	-	-	-	AM20-3, KP899155	
Luteibacter anthropi , FM212516	98.3	43	-	-	-	-	-	-	-	-	4.0	6.7	-	4.5	3.2	3.9	1.6	-	K20-81, KP899192	
Luteibacter rhizovicinus , AJ580498	99.6	44	-	1.0	-	-	-	-	-	-	9.3	8.3	8.2	-	1.6	6.4	1.6	1.4	AK20-111, KP899142	
Luteibacter rhizovicinus , AJ580498	98.1	45	-	-	-	-	-	-	-	-	-	-	-	1.5	-	1.3	1.6	-	AR20-92, KP899172	
Stenotrophomonas maltophilia , AB294553	99.2	46	-	-	-	-	-	-	-	-	1.3	3.3	-	-	-	-	-	-	K20-48, KP899188	
Lysobacter ximonensis , EU237492	97.0	47	-	-	-	-	-	-	-	-	-	-	-	-	1.6	-	-	-	AM20-91, KP899165	
Serratia proteamaculans , AJ233434	99.9	48	4.9	2.0	-	-	3.2	2.5	-	-	1.3	3.3	-	-	-	-	-	1.4	K20-49, KP899189	
Serratia marcescens , AJ233431	96.5	49	-	1.0	-	-	-	-	-	-	-	-	-	-	-	-	-	1.4	K0-89, KP899182	
Perlucidibaca piscinae , DQ664237	97.0	50	-	-	-	-	-	-	-	-	-	-	3.3	3.0	-	-	-	-	S20-49, KP899244	
Pseudomonas constantinii , AF374472	99.3	51	4.9	7.9	-	8.5	3.2	8.9	7.0	9.6	-	1.7	-	-	1.6	1.3	1.6	5.6	K0-92, KP899183	
Pseudomonas constantinii , AF374472	98.5	52	-	11.9	-	-	-	25.3	-	-	-	5.0	-	-	1.6	5.1	1.6	-	K0-67, KP899180	
Pseudomonas lini , AY035996	99.4	53	29.3	32.7	10.1	29.8	14.3	22.8	18.6	19.3	8.0	8.3	4.9	7.5	-	2.6	9.5	5.6	M0-107, KP899198	
Pseudomonas lini , AY035996	98.4	54	9.8	7.9	1.5	2.1	14.3	8.9	-	2.4	4.0	1.7	1.6	7.5	-	6.4	1.6	11.3	K0-71, KP899181	
Pseudomonas psychrophila , AB041885	99.6	55	-	5.0	-	1.1	-	-	-	-	-	3.3	-	-	-	-	-	-	K20-57, KP899191	
Pseudomonas japonica , AB126621	98.8	56	-	-	-	-	-	-	-	-	-	-	-	-	-	-	1.6	-	AR20-23, KP899169	
Pseudomonas lutea , AY364537	97.6	57	-	-	-	1.1	-	-	2.3	-	-	-	-	-	-	-	-	-	S0-17, KP899229	
Pseudomonas jessenii , AF068259	99.9	58	11.0	3.0	-	5.3	38.1	21.5	2.3	10.8	-	5.0	1.6	-	1.6	1.3	-	9.9	K20-95, KP899195	
Pseudomonas jessenii , AF068259	98.4	59	-	-	36.2	3.2	-	-	19.8	19.3	2.7	1.7	3.3	-	-	-	1.6	11.3	R0-91, KP899216	
Chitinophaga arvensicola , AM237311	98.8	60	-	-	-	-	-	-	-	-	-	-	-	-	-	1.3	-	-	AK20-12, KP899143	*Sphingobacteriia*
Chitinophaga costaii , KC922450	98.7	61	-	-	-	-	-	-	-	-	-	-	-	-	1.6	-	-	-	AM20-66, KP899159	
Chitinophaga oryziterrae , JF710262	98.7	62	-	-	-	-	-	-	-	-	-	-	-	-	-	-	-	1.4	AS20-54, KP899176	
Taibaiella smilacinae , KC571459	97.9	63	-	-	-	-	-	-	-	-	1.3	-	-	1.5	-	-	-	-	S20-32, KP899240	
Mucilaginibacter rigui , EU747841	99.3	64	-	-	-	-	-	-	-	-	-	1.7	-	3.0	-	-	-	-	S20-42, KP899242	
Mucilaginibacter lutimaris , HQ455786	96.3	65	-	-	-	-	-	-	-	-	-	-	1.6	-	-	-	-	-	R20-68, KP899226	
Mucilaginibacter dorajii , GU139697	97.4	66	1.2	-	-	-	-	-	-	-	1.3	-	-	-	-	-	-	-	M0-13, KP899200	
Mucilaginibacter boryungensis , HM061614	97.6	67	-	-	-	-	-	-	-	-	1.3	-	1.6	-	-	1.3	-	1.4	R20-71, KP899227	
Mucilaginibacter sabulilitoris , JQ739458	97.5	68	-	-	-	-	-	-	-	-	-	-	3.3	6.0	-	-	-	-	S20-55, KP899245	
Mucilaginibacter lappiensis , DQ234446	98.6	69	1.2	-	-	-	-	-	-	-	1.3	-	-	1.5	3.2	-	-	-	S20-97, KP899252	
Mucilaginibacter lappiensis , DQ234446	98.1	70	-	-	-	-	-	-	-	-	2.7	5.0	-	-	-	-	-	-	K20-24, KP899185	
Mucilaginibacter flavus , HQ449707	97.6	71	2.4	1.0	-	-	-	-	-	-	2.7	-	4.9	3.0	-	-	-	-	M20-56, KP899210	
Mucilaginibacter frigoritolerans , FN400860	96.5	72	-	-	-	-	-	-	-	-	1.3	-	1.6	1.5	-	-	-	-	M20-62, KP899211	
Mucilaginibacter gracilis , AM490403	97.3	73	-	-	-	-	-	-	-	-	-	-	-	1.5	-	-	-	1.4	S20-104, KP899232	
Mucilaginibacter paludis , AM490402	97.1	74	-	-	-	-	-	-	-	-	-	-	-	1.5	-	-	-	-	S20-46, KP899243	
Mucilaginibacter jinjuensis , JQ765855	97.4	75	-	-	-	-	-	-	-	-	-	-	-	-	-	-	4.8	-	AR20-97, KP899174	
Pedobacter nutrimenti , HF536497	99.6	76	4.9	3.0	1.5	-	1.6	-	1.2	-	-	-	-	-	-	-	-	-	M0-12, KP899199	
Pedobacter ginsengisoli, AB245371	98.0	77	1.2	-	-	-	3.2	-	-	1.2	-	-	-	-	-	-	-	-	M0-94, KP899202	
Pedobacter cryoconitis, AJ438170	99.9	78	-	-	18.8	2.1	-	-	4.7	-	-	-	3.3	-	-	-	-	-	R20-57, KP899225	
Pedobacter antarcticus, HM448033	98.6	79	2.4	-	-	-	-	-	-	-	-	-	4.9	1.5	-	-	-	2.8	S20-13, KP899236	
Pedobacter sandarakinus, DQ235228	98.0	80	1.2	-	-	-	-	-	-	-	1.3	-	-	-	-	-	-	1.4	M0-57, KP899201	
Dyadobacter koreensis , EF017660	97.6	81	-	-	-	-	-	-	-	-	1.3	-	-	-	-	-	-	-	M20-49, KP899207	*Cytophagia*
Flavobacterium saccharophilum , AM230491	98.3	82	11.0	8.9	5.8	23.4	-	5.1	3.5	16.9	-	-	1.6	1.5	-	-	-	-	R20-110, KP899218	*Flavobacteriia*
Flavobacterium aquidurense , AM177392	99.0	83	1.2	-	-	-	-	-	-	-	1.3	-	-	-	-	-	-	-	M20-74, KP899213	
Flavobacterium piscis , HE612101	98.1	84	-	1.0	7.3	4.3	-	2.5	3.5	-	-	1.7	-	-	-	-	-	2.8	S0-101, KP899228	
Flavobacterium reichenbachii , AM177616	97.9	85	-	-	-	-	-	-	-	-	-	-	-	1.5	-	-	-	-	S20-105, KP899233	
Flavobacterium hydatis , M58764	98.1	86	-	-	-	2.1	-	-	-	-	4.0	1.7	-	-	-	-	4.8	-	K20-25, KP899186	
Flavobacterium hibernum , L39067	97.4	87	-	-	-	-	-	-	-	-	-	-	-	-	-	-	-	4.2	AS20-23, KP899175	
Flavobacterium succinicans , AM230492	98.7	88	1.2	-	-	-	-	-	-	-	-	1.7	-	-	-	-	-	-	K20-55, KP899190	
Flavobacterium hauense , JX066803	97.3	89	-	-	-	-	-	-	-	-	-	-	-	-	1.6	-	-	-	AM20-97, KP899168	
Chryseobacterium taihuense , JQ283114	97.9	90	-	1.0	-	-	-	-	-	-	1.3	-	-	-	-	-	-	-	K0-43, KP899178	
Plantibacter flavus , AJ310417	98.7	91	-	-	-	-	-	-	-	-	-	-	-	-	-	1.3	-	-	AK20-4, KP899147	*Actinobacteria*
Leifsonia kafniensis , AM889135	98.6	92	-	-	-	-	-	-	-	-	-	-	-	1.5	-	-	-	-	S20-63, KP899246	
Leifsonia poae , AF116342	99.9	93	-	-	-	-	-	-	-	-	-	1.7	-	-	4.8	1.3	-	-	K20-98, KP899196	
Diaminobutyricibacter tongyongensis , JX876865	99.3	94	-	-	-	-	-	-	-	-	2.7	-	-	-	-	1.3	-	1.4	AK20-22, KP899145	
Frondihabitans peucedani , FM998017	99.2	95	-	-	-	-	-	-	-	-	-	-	-	1.5	-	1.3	-	-	S20-31, KP899239	
Arthrobacter psychrochitiniphilus , AB588633	99.5	96	-	-	-	-	-	-	-	-	-	-	-	1.5	-	-	-	-	S20-102, KP899231	
Arthrobacter alpinus , GQ227413	98.6	97	1.2	2.0	-	-	-	-	-	-	-	-	-	-	-	-	-	-	K0-101, KP899177	
Arthrobacter russicus , AB071950	96.1	98	-	-	-	-	-	-	-	-	-	-	-	-	1.6	2.6	-	-	AK20-18, KP899144	
Flexivirga alba , AB539735	99.5	99	-	-	-	-	-	-	-	-	1.3	-	-	-	-	-	-	-	M20-45, KP899206	
Marmoricola aurantiacus , Y18629	96.9	100	-	-	-	-	-	-	-	-	-	-	-	3.0	-	-	-	-	S20-100, KP899230	
Nakamurella multipartita , Y08541	96.2	101	-	-	-	-	-	-	-	-	-	-	-	1.5	-	-	-	-	S20-107, KP899234	
Paenibacillus frigoriresistens , JQ314346	95.3	102	-	-	-	-	-	-	-	-	-	-	1.6	-	-	-	-	-	R20-25, KP899220	*Bacilli*
Paenibacillus pectinilyticus , EU391157	99.4	103	-	-	-	-	-	-	-	-	-	-	-	-	1.6	-	-	-	AM20-37, KP899156	
Paenibacillus pectinilyticus , EU391157	98.4	104	-	-	-	-	-	-	-	-	-	-	-	-	1.6	-	-	-	AM20-92, KP899166	
Paenibacillus graminis , AJ223987	97.6	105	-	-	-	-	-	-	-	-	-	-	3.3	-	-	1.3	1.6	-	R20-18, KP899219	
Paenibacillus wynnii , AJ633647	96.9	106	-	-	-	-	-	-	-	-	-	-	-	1.5	-	-	-	-	S20-76, KP899248	
Paenibacillus odorifer , AJ223990	99.0	107	-	-	-	-	-	-	-	-	-	-	-	1.5	-	-	-	-	S20-92, KP899251	
Paenibacillus granivorans , AF237682	96.8	108	-	-	-	-	-	-	-	-	-	-	-	-	-	-	1.6	-	AR20-56, KP899170	
Paenibacillus tundrae , EU558284	99.0	109	-	-	-	-	-	-	-	-	2.7	-	-	-	-	-	-	-	M20-19, KP899204	
Psychrobacillus psychrodurans , AJ277984	98.9	110	-	-	-	-	-	-	-	-	-	-	1.6	-	-	-	-	-	R20-42, KP899224	
Sporosarcina globispora , X68415	99.9	111	1.2	2.0	-	-	-	-	-	-	-	-	-	-	-	-	-	-	M0-105, KP899197	
Bacillus weihenstephanensis , AB021199	99.9	112	-	-	-	-	-	-	-	-	-	-	-	-	-	-	1.6	1.4	AR20-61, KP899171	

Table S2 Relative abundance (%) of the yeast strains isolated at 4°C and 20°C in spring and autumn. Both the taxonomic affilation and the similarity value to the closest type strains sequence in GenBank database are shown. The abundance is presented in gradient color according to percentages of the total number of isolates in a subset of data. Closest relative identity values below 98% are marked in red.

			4 °C								20 °C										
			Spring				Autumn				Spring				Autumn					Philogenetic affilation	
Closest Type strain, accession number	relative identity (%)	Species	M4 (n=99)	K4 (n=107	R4 (n=50)	S4 (n=68)	AM4 (n=40)	AK4 (n=62)	AR4 (n=46)	AS4 (n=55)	M20 (n=36)	K20 (n=33)	R20 (n=18)	S20 (n=43)	AM20 (n=6)	AK20 (n=23)	AR20 (n=13)	AS20 (n=20)	Representative isolate, accession number	Order	Classes
Aureobasidium pullulans, FJ150902	100.0	1	-	2.80	-	-	-	-	-	-	-	6.06	-	-	-	-	-	-	DBVPG10530, KU745308	*Dothideomycetes*	*Dothideales*
Babjeviella inositovora, NR111018	99.1	2	-	-	-	-	-	-	-	-	-	-	-	-	-	-	7.69	-	DBVPG10531, KU745309	Saccharomycetales	Saccharomycetes
Candida oregonensis, HM156520	99.6	3	-	-	-	-	-	-	2.17	-	-	-	-	-	-	-	-	-	DBVPG10533, KU745311		
Candida santamariae, AY542869	100.0	4	-	2.80	-	-	-	1.61	-	-	-	21.21	-	-	-	-	-	-	DBVPG10534, KU745312		
Candida santamariae, AY542869	99.8	5	-	-	-	-	-	-	-	-	-	6.06	-	-	-	-	-	-	DBVPG10535, KU745313		
Candida santamariae, AY542869	99.2	6	-	-	-	-	-	-	-	-	-	-	-	-	-	-	7.69	-	DBVPG10536, KU745314		
Candida santamariae, AY542869	99.0	7	-	-	-	-	-	-	2.17	1.82	-	-	-	-	-	-	7.69	-	DBVPG10537, KU745315		
Debaryomyces hansenii KM091320	99.1	8	-	-	-	-	-	-	-	-	2.78	-	-	-	-	-	-	-	DBVPG10584, KU745355		
Schizoblastosporion starkeyihenricii, CBS2159NT	99.8	9	-	-	-	1.47	-	-	-	-	-	-	16.67	-	-	-	-	-	DBVPG10603, KU745373		
Piskurozyma sp., HG324303[a]	88.1	10	-	-	-	-	-	-	-	-	-	-	-	-	-	-	-	5.00	DBVPG10557, KU745333	Filobasidiales	Tremellomycetes
Filobasidium sp, NR130665[b]	99.0	11	-	-	-	-	-	-	-	-	-	-	-	2.33	-	-	-	-	DBVPG10556, KU745332		
Goffeauzyma gastrica, NR111048	100.0	12	-	1.87	4.00	4.41	-	-	-	-	-	-	-	-	-	-	-	-	DBVPG10542, KU745319		
Goffeauzyma gastrica, NR111048	100.0	13	-	-	-	-	-	-	4.35	-	-	-	-	-	-	4.35	-	5.00	DBVPG10543, KU745320		
Naganishia albida, NR111046	99.8	14	1.01	-	-	-	-	-	-	-	-	-	-	-	-	-	-	-	DBVPG10539, KU745316		
Naganishia diffluens, NR111051	100.0	15	-	-	-	-	-	-	-	-	-	-	5.56	-	-	-	-	-	DBVPG10540, KU745317		
Solicoccozyma fuscescens, AF145319	100.0	16	-	-	-	-	-	-	-	-	-	-	-	2.33	-	-	-	-	DBVPG10541, KU745318		
Solicoccozyma phenolicus, NR073222	100.0	17	-	-	-	1.47	-	-	-	-	-	-	-	-	-	-	-	-	DBVPG10545, KU745322		
Solicoccozyma phenolicus, NR073222[c]	99.4	18	-	-	-	-	2.50	-	-	-	-	-	-	-	-	-	-	-	DBVPG10558, KU745334		
Solicoccozyma terreus, NR073212[d]	99.8	19	1.01	-	2.00	2.94	-	-	-	-	-	-	-	-	-	-	-	-	DBVPG10559, KU745335		
Solicoccozyma terreus, NR073212[e]	99.8	20	-	-	-	-	-	-	-	-	-	-	-	-	16.67	-	-	5.00	DBVPG10560, KU745336		
Solicoccozyma terreus, NR073212[f]	99.6	21	-	-	-	-	-	-	-	-	-	-	-	2.33	-	-	-	-	DBVPG10561, KU745337		
Solicoccozyma terricola, NR073221	100.0	22	23.23	16.82	52.00	55.88	-	-	-	-	25.00	3.03	27.78	30.23	-	-	-	-	DBVPG10562, KU745338		
Solicoccozyma terricola, NR073221	100.0	23	-	-	-	-	52.50	29.03	32.61	36.36	-	-	-	-	16.67	-	23.08	20.00	DBVPG10563, KU745339		
Solicoccozyma terricola, NR073221	99.8	24	3.03	3.74	8.00	-	17.50	1.61	-	5.45	-	6.06	-	2.33	-	8.70	-	-	DBVPG10564, KU745340		
Solicoccozyma terricola, NR073221	99.7	25	2.02	0.93	-	-	-	-	2.17	-	-	-	-	2.33	-	-	-	-	DBVPG10565, KU745341		
Solicoccozyma terricola, NR073221	99.6	26	1.01	-	-	-	2.50	1.61	-	-	2.78	-	-	-	-	-	-	-	DBVPG10566, KU745342		
Solicoccozyma terricola, NR073221	99.5	27	-	2.80	-	1.47	-	-	-	-	-	-	-	-	-	-	-	-	DBVPG10567, KU745343		
Solicoccozyma terricola, NR073221	99.3	28	-	2.80	-	-	-	1.61	-	-	-	-	-	-	-	-	-	-	DBVPG10569, KU745344		
Solicoccozyma terricola, NR073221	98.8	29	-	-	-	-	-	-	4.35	-	-	-	-	-	-	-	7.69	5.00	DBVPG10572, KU745345		
Solicoccozyma terricola, NR073221	98.6	30	-	-	2.00	1.47	-	-	-	3.64	-	-	-	-	-	-	-	-	DBVPG10573, KU745346		
Holtermanniella festucosa, AY462120	100.0	31	-	-	-	-	-	-	2.17	-	-	-	-	-	-	-	-	-	DBVPG10588, KU745359	*Holtermanniales*	
Holtermanniella wattica, FJ473373	99.7	32	-	-	-	1.47	-	-	-	-	-	-	-	-	-	-	-	-	DBVPG10589, KU745360		
Holtermanniella wattica, FJ473373	99.7	33	-	-	-	-	-	-	-	1.82	-	-	-	-	-	-	-	-	DBVPG10590, KU745361		
Holtermanniella wattica, FJ473373[g]	99.7	34	-	-	-	-	-	-	-	-	-	-	-	2.33	-	-	-	-	DBVPG10591, KU745362		
Dioszegiahungarica, NR073227	99.5	35	1.01	-	-	-	-	-	-	-	-	-	-	-	-	-	-	-	DBVPG10585, KU745356	Tremellales	
Vishniacozyma victoriae, NR073260	99.6	36	-	-	-	-	-	1.61	-	-	-	-	-	-	-	-	-	-	DBVPG10576, KU745347		
Vishniacozyma victoriae, NR073260	99.5	37	1.01	-	-	-	-	1.61	-	-	-	-	-	-	-	-	-	-	DBVPG10577, KU745348		
Bullera alba, NG042387	100.0	38	-	-	-	-	2.50	-	-	-	-	-	-	-	-	-	-	-	DBVPG10532, KU745310		
Saitozyma podzolica, NR073213	100.0	39	-	-	-	-	-	-	-	-	5.56	9.09	-	23.26	-	-	-	-	DBVPG10546, KU745323		
Saitozyma podzolica, NR073213	99.8	40	-	-	-	2.94	-	-	-	-	2.78	-	27.78	20.93	-	17.39	-	20.00	DBVPG10548, KU745325		
Saitozyma podzolica, NR073213	99.5	41	1.01	-	-	1.47	-	-	-	-	8.33	9.09	-	-	-	-	-	-	DBVPG10549, KU745326		
Saitozyma podzolica, NR073213	99.5	42	-	-	-	-	-	3.22	-	-	-	-	-	-	16.67	13.04	-	5.00	DBVPG10550, KU745327		
Saitozyma podzolica, NR073213	99.3	43	-	-	-	-	-	-	-	1.82	-	-	-	2.33	-	-	-	5.00	DBVPG10552, KU745329		
Saitozyma podzolica, NR073213	99.1	44	-	-	-	-	-	-	-	-	-	-	-	2.33	33.33	-	-	-	DBVPG10553, KU745330		
Apiotrichum porosum, NR073209	100.0	45	41.41	55.13	24.00	22.06	-	-	-	-	36.11	39.39	16.67	4.65	-	-	-	-	DBVPG10607, KU745377	Trichosporonales	
Apiotrichum porosum, NR073209	100.0	46	-	-	-	-	17.50	53.23	28.26	29.09	-	-	-	-	-	43.48	30.77	20.00	DBVPG10608, KU745378		
Apiotrichum porosum, NR073209	99.3	47	-	0.93	-	1.47	-	-	2.17	-	-	-	-	-	-	-	-	-	DBVPG10611, KU745379		
Apiotrichum porosum, NR073209	99.2	48	-	0.93	-	-	-	-	2.17	-	-	-	-	-	-	-	-	-	DBVPG10612, KU745380		
Cutaneotrichosporon moniliiforme, NR073240	99.8	49	3.03	0.93	-	-	2.50	1.61	-	-	2.78	-	-	-	16.67	-	-	-	DBVPG10605, KU745375		
Cutaneotrichosporon moniliiforme, NR073240	99.7	50	1.01	-	-	-	-	-	-	-	-	-	-	-	-	-	-	-	DBVPG10606, KU745376		
Vanrija musci, AB035579[h]	100.0	51	-	-	-	-	-	-	-	-	-	-	-	-	-	-	7.69	-	DBVPG10544, KU745321		
Cystofilobasidium capitatum, NR111042	100.0	52	2.02	4.67	-	1.47	-	-	-	-	-	-	-	-	-	-	-	-	DBVPG10579, KU745350	Cystofilobasidiales	
Cystofilobasidium capitatum, NR111042	100.0	53	-	-	-	-	2.50	1.61	4.35	-	-	-	-	-	-	-	-	-	DBVPG10580, KU745351		
Cystofilobasidium capitatum, NR111042	99.8	54	1.01	-	-	-	-	-	-	-	-	-	-	-	-	-	-	-	DBVPG10581, KU745352		
Cystofilobasidium capitatum, NR111042	99.8	55	-	-	-	-	-	-	-	1.82	-	-	-	-	-	-	-	-	DBVPG10582, KU745353		
Cystofilobasidum macerans, EU082231	97.2	56	-	-	-	-	-	-	-	1.82	-	-	-	-	-	-	-	-	DBVPG10583, KU745354		
Itersonilia pannonica, NR077110	100.0	57	2.02	-	-	-	-	-	-	-	-	-	-	-	-	-	-	-	DBVPG10617, KU745381		
Krasilnikovozyma sp., KF036599[i]	81.0	58	1.01	-	-	-	-	-	-	-	-	-	-	-	-	-	-	-	DBVPG10597, KU745368		
Mrakia gelida, AF144485	100.0	59	-	-	2.00	-	-	-	-	-	-	-	-	-	-	-	-	-	DBVPG10594, KU745365		
Mrakia gelida, AF144485	100.0	60	-	-	-	-	-	-	4.35	1.82	-	-	-	-	-	-	-	-	DBVPG10595, KU745366		
Mrakia gelida, AF144485	99.7	61	-	-	-	-	-	-	2.17	-	-	-	-	-	-	-	-	-	DBVPG10596, KU745367		
Tausonia pullulans, AF444417	100.0	62	-	-	4.00	-	-	-	-	-	-	-	5.56	-	-	-	-	-	DBVPG10586, KU745357		
Tausonia pullulans, AF444417	100.0	63	-	-	-	-	-	-	4.35	3.64	-	-	-	-	-	-	-	-	DBVPG10587, KU745358		
Rhodosporidiobolus colostri, HF558663	100.0	64	6.06	2.80	-	-	-	1.61	-	-	-	-	-	-	-	-	-	-	DBVPG10599, KU745369	*Sporidiobolales*	Microbotryomycetes
Rhodosporidiobolus colostri, HF558663	99.8	65	-	-	-	-	-	-	-	1.82	-	-	-	-	-	-	-	-	DBVPG10600, KU745370		
Sporobolomyces roseus, AB030351	100.0	66	1.01	-	-	-	-	-	-		8.33	-	-	-	-	-	-	-	DBVPG10604, KU745374		
Leucosporidium drummii, FN908919	100.0	67	-	-	2.00	-	-	-	-	-	-	-	-	2.33	-	-	-	-	DBVPG10592, KU745363	*Leucosporidiales*	
Leucosporidium drummii, FN908919	100.0	68	-	-	-	-	-	-	2.17	9.09	-	-	-	-	-	-	7.69	-	DBVPG10593, KU745364		
Yamadamyces rosulatus, EU872492	100.0	69	-	-	-	-	-	-	-	-	-	-	-	-	-	-	-	5.00	DBVPG10601, KU745371	*Kriegeriales*	
Curvibasidum pallidicorallinum, AF444641	100.0	70	7.07	-	-	-	-	-	-	-	5.56	-	-	-	-	-	-	-	DBVPG10578, KU745349	*Incertae sedis*	
Rhodotorula sp, NR073305[l]	90.0	71	-	-	-	-	-	-	-	-	-	-	-	-	-	-	-	5.00	DBVPG10602, KU745372		

Closest relative D1/D2 of the 26S rRNA gene accession numbers: a, HG324303; b, KC433838; c, AF181523; d,AF075479; e, AF075479; f, AF075479; g, KC433858; h, AB126586; i, EF118826; l, FN428969

Representative isolate D1/D2 of the 26S rRNA gene accession numbers: a, KU745299; b, KU745298; c, KU745300; d, KU745301; e, KU745302; f, KU745303; g, KU745305; h, KU745297; i, KU745306; l, KU745307
